# Targeting melanocortin 4 receptor to treat sleep-disordered breathing in mice

**DOI:** 10.1172/JCI177823

**Published:** 2025-04-15

**Authors:** Mateus R. Amorim, Noah R. Williams, O. Aung, Melanie Alexis Ruiz, Frederick Anokye-Danso, Junia L. de Deus, Jiali Xiong, Olga Dergacheva, Shannon Bevans-Fonti, Sean M. Lee, Jeffrey S. Berger, Mark N. Wu, Rexford S. Ahima, David Mendelowitz, Vsevolod Y. Polotsky

**Affiliations:** 1 Department of Anesthesiology and Critical Care Medicine, George Washington University, Washington, DC, USA.; 2 Department of Physiology, Medical School of Ribeirão Preto, Sao Paulo, Brazil.; 3 Medical College of Wisconsin, Milwaukee, Wisconsin, USA.; 4 Department of Medicine, Johns Hopkins University, Baltimore, Maryland, USA.; 5 Biochemistry, Cellular, and Molecular Biology Graduate Program, Johns Hopkins University School of Medicine, Baltimore, Maryland, USA.; 6 Department of Pharmacology and Physiology and; 7 Office of Clinical Research, George Washington University, Washington, DC, USA.; 8 Department of Neurology and; 9 Division of Endocrinology, Diabetes and Metabolism, Department of Medicine, Johns Hopkins University School of Medicine, Baltimore, Maryland, USA.; 10 Department of Medicine, George Washington University, Washington, DC, USA.

**Keywords:** Neuroscience, Pulmonology, Mouse models, Obesity, Pharmacology

## Abstract

Weight loss medications are emerging candidates for pharmacotherapy of sleep-disordered breathing (SDB). A melanocortin 4 receptor (MC4R) agonist, setmelanotide (Set), is used to treat obesity caused by abnormal melanocortin and leptin signaling. We hypothesized that Set can treat SDB in mice with diet-induced obesity. We performed a proof-of-concept randomized crossover trial of a single dose of Set versus vehicle and a 2-week daily Set versus vehicle trial, examined colocalization of *Mc4r* mRNAs with the markers of CO_2_-sensing neurons *Phox2b* and neuromedin B in the brainstem, and expressed *Cre*-dependent designer receptors exclusively activated by designer drugs (DREADDs) or caspase in obese *Mc4r-Cre* mice. Set increased minute ventilation across sleep/wake states, enhanced the hypercapnic ventilatory response (HCVR), and abolished apneas during sleep. *Phox2b*^+^ neurons in the nucleus of the solitary tract (NTS) and the parafacial region expressed *Mc4r*. Chemogenetic stimulation of the MC4R^+^ neurons in the parafacial region, but not in the NTS, augmented HCVR without any changes in metabolism. Caspase elimination of the parafacial MC4R^+^ neurons abolished effects of Set on HCVR. Parafacial MC4R^+^ neurons projected to the respiratory premotor neurons retrogradely labeled from C3–C4. In conclusion, MC4R agonists enhance the HCVR and treat SDB by acting on the parafacial MC4R^+^ neurons.

## Abstract 2

Amorim et al., show that setmelanotide enhance the hypercapnic ventilatory response and treat sleep disordered breathing by acting on the parafacial MC4R+ neurons.

## Graphical abstract

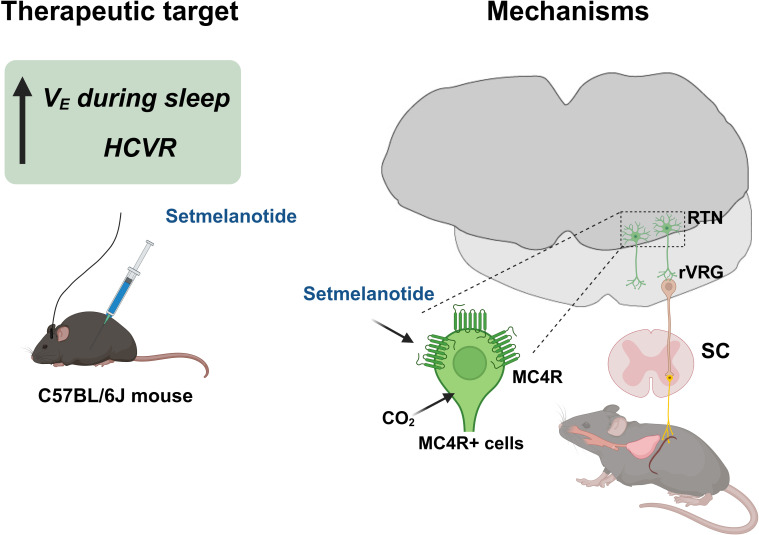

## Introduction

Obesity hypoventilation syndrome (OHS) is a life-threatening severe form of sleep-disordered breathing (SDB) characterized by daytime hypercapnia and hypoventilation during sleep. OHS is observed in 10%–20% of obese patients with obstructive sleep apnea (OSA) (1, 2), recurrent upper airway obstruction during sleep (3, 4). The prevalence of OSA in obesity exceeds 50% (5–10). Although the exact prevalence of OHS is unknown, given that about 35% of American adults (11), or 90 million people, are obese, approximately 5 to 10 million US adults have OHS. Untreated or suboptimally treated OHS leads to high morbidity and mortality (1) with an all-cause mortality of 18% at 1 year, 24% at 1.5 years (12), and 31.3% at 3 years of follow-up (13, 14). Treatment of OHS consists of treatment of upper airway obstruction during sleep with continuous positive airway pressure (CPAP) and noninvasive ventilation with bilevel positive airway pressure (BiPAP) (15, 16). However, poor adherence with CPAP and BiPAP limits their effectiveness (17, 18). There is no effective pharmacotherapy for OHS.

OHS is driven by decreased CO_2_ sensitivity and depressed ventilatory responses to CO_2_ (hypercapnic ventilatory response [HCVR]) (1, 19–21). One promising direction of OHS therapy is augmentation of HCVR. Several groups of investigators have previously shown that the adipose-produced hormone leptin, which suppresses food intake and increases metabolic rate (22–25), also augments hypercapnic sensitivity and treats hypoventilation in leptin-deficient *ob/ob* mice (26, 27). We have shown that mice with diet-induced obesity (DIO mice) emulate all features of human OHS, including awake hypercapnia, upper airway obstruction during sleep, sleep hypoventilation, high plasma leptin levels, and leptin resistance (28–31). We have also shown that resistance to intravenous and intraperitoneal leptin treatment in DIO mice can be overcome by intranasal leptin administration (28, 32). However, leptin resistance is a major obstacle to using leptin therapeutically, and this resistance is mediated by multiple mechanisms (33–37), including limited permeability of the blood-brain barrier (38, 39). Targeting CNS sites downstream of leptin provides an opportunity to bypass leptin resistance and identify targets to augment hypercapnic sensitivity and treat SDB.

Leptin activates anorexigenic proopiomelanocortin (POMC) neurons of the arcuate nucleus and increases POMC expression (40–42). POMC is posttranslationally processed to several peptides, including α-melanocyte-stimulating hormone (α-MSH) (43). α-MSH is a natural ligand for the melanocortin 4 receptor (MC4R), a G protein–coupled receptor, a critical regulator of food intake and energy expenditure. MC4R is expressed throughout the brain, especially in metabolic centers of the hypothalamus (44, 45) and, in the brainstem, in the autonomic nervous system centers of medulla, such as the nucleus of the solitary tract (NTS) and rostral ventrolateral medulla (44, 45). MC4R blockade induces obesity in rodents, e.g., in mice overexpressing a natural MC4R blocker, an agouti-related peptide (AgRP) (24, 46); MC4R-knockout mice also show an obesity phenotype (47). Mutations in the MC4R pathways are the most common single-gene disorders leading to human obesity with a prevalence of 1% to 6% in the obese population, and are especially common in severe childhood-onset obesity (48–50). Moreover, an MC4R agonist, setmelanotide (Set), which has a 20-fold MC4R selectivity compared with α-MSH (51), has been effective for weight loss in patients with the MC4R/POMC/leptin pathway polymorphism and has been approved by the FDA for treatment of obesity in these patients (50, 52–54). Set is an MC4R/MC3R agonist with 15- to 20-fold less activity at the melanocortin 3 receptor (50). In contrast to other MC4R agonists, Set does not cause tachycardia or hypertension (51, 55, 56). The potential benefits of Set for treating OHS have not yet been tested.

The discovery of respiratory effects of leptin and leptin resistance encouraged investigators to explore whether the MC4R pathway can have a stimulatory effect on control of breathing. We have shown that A^y^ mice overexpressing the natural MC4R blocker AgRP hypoventilate during sleep and have suppressed HCVR compared with weight-matched wild-type control mice (57). Bassi et al. showed that intracerebroventricular injections of the MC4R blocker SHU9119 decreased ventilatory responses to 8% CO_2_ and attenuated leptin-induced hyperventilation in rats (58). Several case reports of severe SDB in obese children with mutations in the *Mc4r* gene have been published (59, 60). A recent study showed that Set increased HCVR in DIO male mice, but a similar effect was observed in pair-fed animals (61). The role of MC4R in control of breathing remains poorly understood, and MC4R agonists have not been tested for SDB treatment. We hypothesized that the MC4R agonist Set would enhance HCVR and treat SDB in DIO mice, that MC4R is expressed in the CO_2_-sensing neurons, and that targeted activation of MC4R in these neurons would enhance hypercapnic sensitivity in DIO.

Our hypothesis was addressed in a series of experiments in mice. First, we performed a randomized controlled crossover trial of a single dose of Set followed by sleep studies, HCVR measurements, and metabolic studies. Second, we performed a randomized placebo-controlled trial of daily Set for 2 weeks. Third, we performed *Mc4r*/*Phox2b* in situ hybridization to examine MC4R colocalization in CO_2_-sensing regions of the brain. *Phox2b* is universally present in CO_2_-sensing neurons of the NTS and the retrotrapezoid nucleus (RTN) (62–66). *Phox2b* mutations lead to loss of CO_2_ sensitivity, causing congenital central hypoventilation syndrome (67). The CO_2_-sensitive neuronal phenotype is also characterized by the absence of tyrosine hydroxylase and choline acetyltransferase and the presence of neuromedin B (*Nmb*) (65, 66, 68, 69), and, thus, MC4R colocalization of these biomarkers was explored. Fourth, based on RNAscope data, we examined the effect of MC4R^+^ neurons on control of breathing in a gain-of-function experiment. For that purpose, we transfected obese *Mc4r-Cre* mice with adeno-associated virus (AAV) carrying *Cre*-dependent excitatory hM3(Gq) designer receptors exclusively activated by designer drugs (DREADDs) to the medullary centers implicated in CO_2_ sensitivity, including the NTS and the parafacial region containing the RTN. The transfected mice were treated with the DREADDs-specific ligand J60 versus saline in a crossover manner followed by HCVR measurements and metabolic studies. Fifth, we examined the effect of MC4R^+^ RTN neurons on control of breathing in a loss-of-function experiment. We transfected obese *Mc4r-Cre* mice with AAV carrying *Cre-*dependent caspase into the RTN and measured the effect of Set on room air ventilation and HCVR before and after caspase transfection. Sixth, we examined anatomic relationships between parafacial MC4R^+^ neurons and respiratory motoneurons. The RTN neurons do not project to the spinal cord, but show distinct projections to the rostral ventral respiratory group (rVRG) neurons (65, 70), which innervate the phrenic nucleus in the spinal cord. In the *Mc4r-Cre* mice, the parafacial region was transfected with the *Cre*-dependent EYFP-expressing virus, while the retrograde tracer cholera toxin B (CTB) was administered to the C3–C4 spinal cord segments, and localization of MC4R^+^ axons and CTB^+^ respiratory premotor neurons in the rVRG was determined.

## Results

### A single dose of Set stimulated breathing during wakefulness out of proportion to increases in metabolism in male mice.

**Figure 1:**
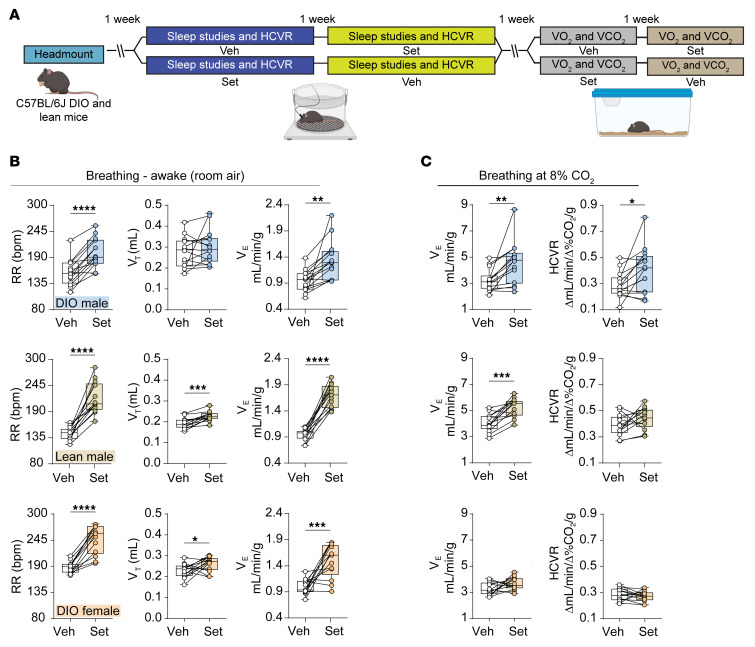
A single dose of Set stimulates breathing and augments the HCVR. (**A**) Experimental design. (**B**) Individual and grouped data show the differences between the effects of vehicle (Veh) and Set on the respiratory rate (RR), tidal volume (*V*_T_), and minute ventilation (*V*_E_) in awake DIO male (top panels, *n* = 14), lean male (middle panels, *n* = 9–13), and DIO female mice (bottom panels, *n* = 10–13) under room air conditions. (**C**) *V*_E_ at 8% of inspired CO_2_ and HCVR. *P ≤ 0.05, **P < 0.01, ***P < 0.001, ****P < 0.0001 using a paired 2-tailed *t* test or Wilcoxon’s matched-pairs, signed-rank test.

In DIO and lean male mice, a single dose of Set increased minute ventilation (*V*_E_) compared with vehicle (Veh) (*P* < 0.05) (Figure 1, A and B). Increases in respiratory rate (RR) were noted in both weight groups, whereas tidal volume (*V*_T_) was significantly increased only in lean mice. In both DIO and lean male mice, Set augmented *V*_E_ during 8% CO_2_ exposure and increased both RR and *V*_T_, whereas a significant increase in the HCVR (*P* < 0.05) was noted only in DIO male animals (Figure 1C, Figure 3A, and Supplemental Figure 1; supplemental material available online with this article; https://doi.org/10.1172/JCI177823DS1). In DIO female mice, Set stimulated baseline *V*_E_ by increasing RR and *V*_T_. Further, Set increased RR under hypercapnic conditions compared with Veh, but did not increase hypercapnic *V*_T_, *V*_E_, or HCVR (Figure 1, B and C, and Supplemental Figure 1).

**Figure 2:**
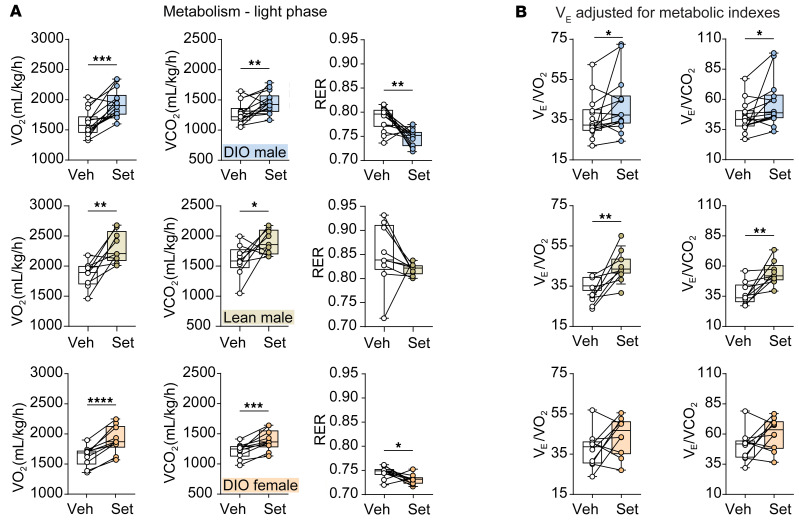
A single dose of Set increases total oxygen consumption (VO_2_) and carbon dioxide production (VCO_2_) compared with Veh, but *V*_E_ increases out of proportion to increases in VO_2_ and VCO_2_. Data in DIO male (top panels, *n* = 14), lean male (middle panels, *n* = 9–13), and DIO female mice (bottom panels, *n* = 10–13) are shown. (**A**) VO_2_, VCO_2_, and respiratory exchange ratio (RER) throughout the light phase. (**B**) *V*_E_/VO_2_ and *V*_E_/VCO_2_. *P < 0.05, **P < 0.01, ***P < 0.001, ****P < 0.0001 using a paired 2-tailed *t* test or Wilcoxon’s matched-pairs, signed-rank test.

Metabolic measurements were performed throughout the light phase (when mice were predominantly asleep and all respiratory data were collected) and the dark phase. During the light phase, a single dose of Set increased O_2_ consumption (VO_2_) and CO_2_ production (VCO_2_) in all studied male and female mice, whereas a decrease in the respiratory exchange ratio was noted only in DIO mice of both sexes, which suggests increased utilization of fat (Figure 2A). Mouse activity did not differ in mice treated with Set versus Veh (Supplemental Figure 2). To determine whether increased ventilation was due to changes in VCO_2_ or VO_2_, we calculated the *V*_E_ adjusted for metabolic indexes. We found that Set increased the *V*_E_/VO_2_ and *V*_E_/VCO_2_ ratios compared with Veh in both DIO and lean males, suggesting that breathing was stimulated out of proportion to the upregulation of metabolism (*P* < 0.05) (Figure 2B). Set did not affect the *V*_E_/VO_2_ and *V*_E_/VCO_2_ ratios in DIO females, suggesting that, in contrast to male animals, Set increases breathing in proportion to increased CO_2_ production in female animals (Figure 2B). VCO_2_ and VO_2_ were not affected by Set during the dark phase, but the respiratory exchange ratio was decreased in relation to Veh in DIO mice of both sexes (Supplemental Figure 2).

### The stimulatory effect of Set on control of breathing persisted during sleep.

Sleep architecture for all groups is described in Table 1. Set reduced sleep efficiency and total sleep time, decreasing both non–rapid eye movement (NREM) and rapid eye movement (REM) sleep time, and induced NREM sleep fragmentation, increasing the number of NREM sleep bouts of shorter duration, in both DIO and lean males. Set did not affect sleep architecture in DIO female mice, which showed low sleep efficiency regardless of treatment.

**Figure 3:**
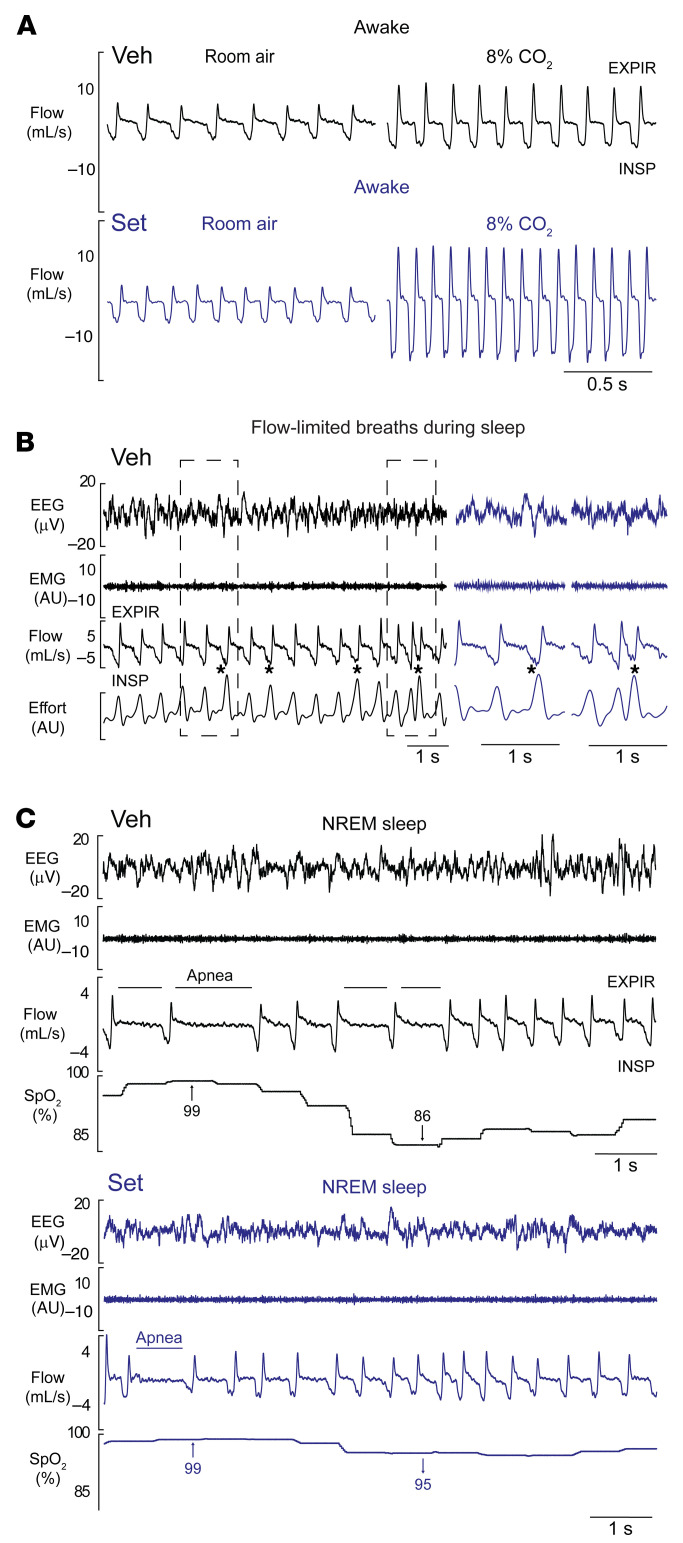
Representative respiratory recordings in DIO male mice treated with a single dose of Set or Veh. (**A**) Set increased minute ventilation in room air, increasing RR, and enhanced the HCVR during wakefulness. (**B**) Obese mice showed inspiratory flow limitation (asterisks) in the presence of increased respiratory effort during sleep, which is a hallmark manifestation of upper airway obstruction. A representative tracing of NREM sleep in a mouse treated with Veh is shown. Outlined fragments on the left are expanded on the right. (**C**) Several central apneas with oxyhemoglobin desaturations during NREM sleep in an obese mouse treated with Veh (left), which significantly improved after Set treatment (right). EEG, electroencephalogram; EMG, nuchal electromyogram; SpO_2_, oxyhemoglobin saturation; INSP, inspiration; EXPIR, expiration. On the respiratory tracings, inspirations are down and expirations are up. a.u., arbitrary units.

We determined each breath’s respiratory characteristics using an algorithm to determine whether a breath was inspiratory flow–limited (IFL) or unobstructed. IFL breaths were characterized by an early inspiratory plateau in airflow at a maximum level (29, 71–73) despite increased effort, a primary feature of upper airway obstruction during sleep in humans with OSA (74–77) (Figure 3B). In contrast, in unobstructed breaths, which are driven by metabolic demands and neural control, the inspiratory limb was rounded in shape without a plateau in mid-inspiration (Figure 3B).

**Figure 4:**
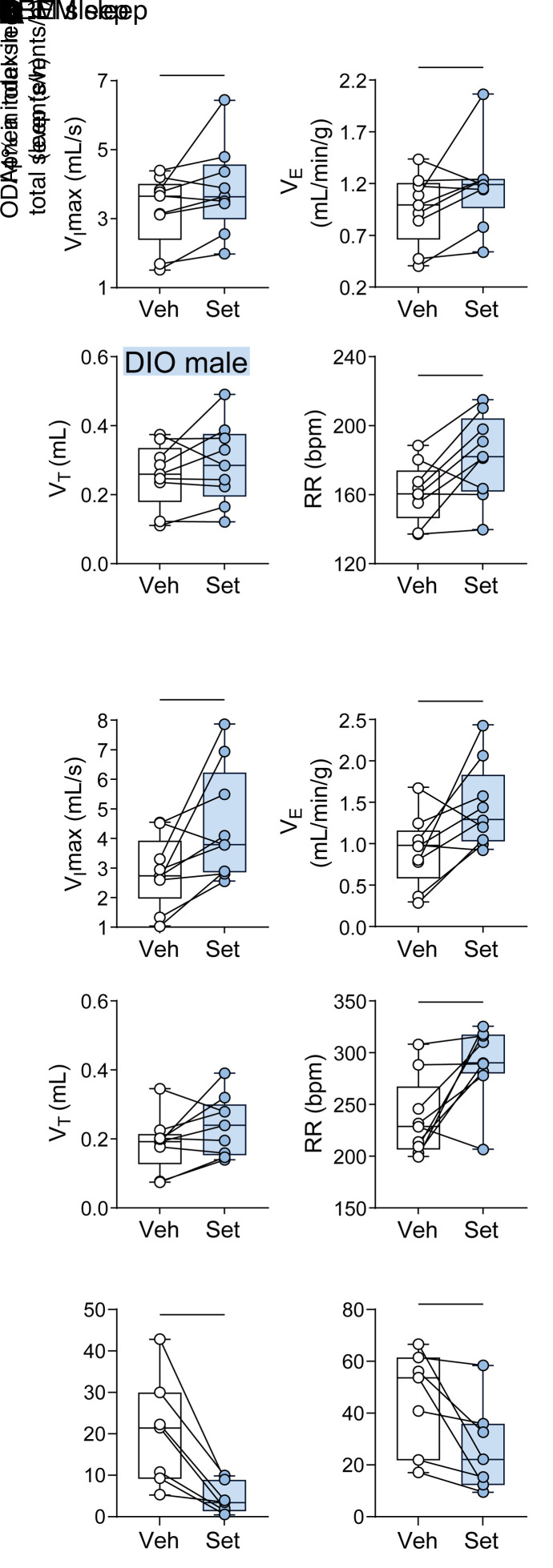
Set increases ventilation during sleep. (**A** and **B**) Sleep studies showed that Set significantly increased maximal inspiratory flow (*V*_I_max), minute ventilation (*V*_E_), and respiratory rate (RR) during NREM (**A**) and REM (**B**) sleep in male DIO mice. (**C** and **D**) Set nearly abolished apneas (**C**) and improved the oxyhemoglobin desaturation index (ODI; ≥4% from pulse oximetry baseline) (**D**). *V*_T_, tidal volume. *n* = 7–9. *P ≤ 0.05 using the Wilcoxon’s matched-pairs, signed-rank test.

Representative polysomnography recording of NREM sleep in a Veh-treated DIO mouse with an apneic event is shown in Figure 3C. The quantitative analysis of unobstructed breathing in DIO male mice showed that Set increased maximal inspiratory flow (*V*_I_max), mean inspiratory flow rate (MIFR), minute ventilation (*V*_E_), and respiratory rate (RR) during both NREM and REM sleep compared with Veh (*P* ≤ 0.05) (Figure 4, Supplemental Figure 3A, and Supplemental Figure 4A). In lean males, Set stimulated unobstructed breathing predominantly in NREM sleep, increasing *V*_I_max, *V*_E_, RR, and MIFR (Supplemental Figure 5A), while in REM sleep it trended to increase RR only (Supplemental Figure 5B). In DIO female mice, Set increased *V*_I_max, RR, and MIFR during unobstructed NREM sleep breathing, but *V*_E_ was not impacted (Supplemental Figure 6). Breathing during REM sleep was not quantified in DIO female mice owing to the low amount of REM sleep regardless of treatment.

The frequency of IFL (or obstructed) breath varied between 10% and 20% of all breaths in NREM sleep and between 20% and 40% in REM sleep, which was consistent with our prior report (29). During IFL breathing in NREM sleep, Set increased inspiratory flow, both *V*_I_max and MIFR, and oxyhemoglobin saturation (SpO_2_) during NREM sleep (Supplemental Figures 3, 5, and 6) in all studied mice. During IFL breathing in REM sleep in DIO male mice, Set significantly increased *V*_I_max and *V*_T_ (Supplemental Figure 4), which suggests that upper airway obstruction decreased.

The longitudinal breath-by-breath analysis showed that Set stimulated breathing throughout the entire sleep period, significantly increasing RR in DIO male mice (Supplemental Figure 7). Our data suggest that Set consistently stimulated breathing throughout sleep/wake states, which was evident from increases in inspiratory flow and *V*_E_ during unobstructed breathing, and relieved upper airway obstruction during sleep, which was evident from increases in *V*_I_max during obstructed breathing. Importantly, Set decreased apneas, which were predominantly central, and oxyhemoglobin desaturation indices during sleep (*P* < 0.05) (Figure 3C, Figure 4, C and D, and Supplemental Figure 8). Thus, a single dose of the MC4R agonist Set stimulated control of breathing, increased *V*_E_ during wakefulness and sleep, and improved upper airway patency during sleep.

### Set treatment for 2 weeks augmented HCVR and decreased the number of apneas during sleep.

**Figure 5:**
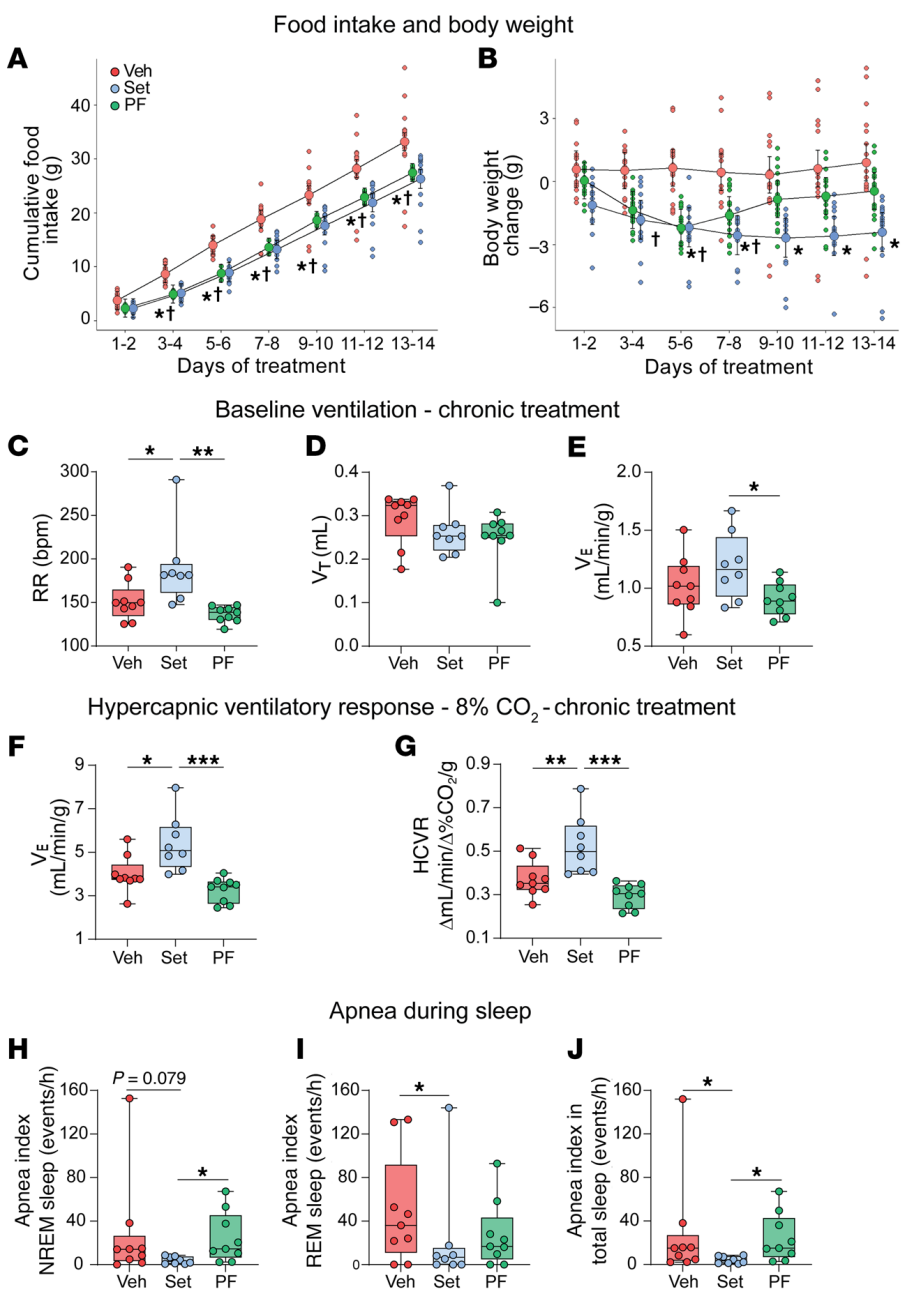
Set treatment for 2 weeks augments the HCVR. (**A**–**E**) Set decreased food intake (**A**), induced mild weight loss (*P < 0.05, Set vs. Veh, ^†^*P* < 0.05, Set vs. pair-fed [PF] using fitted linear mixed models and Tukey’s multiple comparison) (**B**), increased RR (**C**) but not *V*_T_ (**D**), and increased *V*_E_ in room air (**E**). (**F** and **G**) Set increased *V*_E_ at 8% CO_2_ (**F**) and HCVR (**G**) in DIO male C57BL/6J mice in comparison with Veh and PF (*P ≤ 0.05, **P < 0.01, ***P < 0.001, using 1-way ANOVA and Tukey’s multiple-comparison test). (**H**–**J**) Set decreased apnea index during NREM (**H**), REM (**I**), and total (**J**) sleep (*P < 0.05 using fitted robust linear models). *n* = 9 in the Set group; *n* = 8 in the Veh group; *n* = 9 in PF group.

Next, we examined whether the effect of Set on breathing persists during chronic treatment. We compared the effects of chronic Set versus Veh and pair feeding for 14 days, examining food intake and body weight progression, respiratory indices, and the HCVR during wakefulness and the apnea rate during sleep in DIO mice. As expected, Set induced modest weight loss (*P* = 0.01) (Figure 5, A and B). Pair-fed mice also showed weight loss within the first 7 days, but then slowly regained weight. Chronic treatment with Set increased baseline RR and room air *V*_E_ (Figure 5, C–E). Set greatly enhanced the HCVR and increased *V*_E_ in 8% CO_2_ compared with the Veh (placebo) and pair-fed groups (*P* < 0.05) (Figure 5, F and G). Specifically, under hypercapnic conditions Set-treated mice had significantly higher RR than mice in both control groups (*P* < 0.001) (Supplemental Figure 9), while *V*_T_ was augmented in comparison with pair-fed animals (*P* < 0.05). Sleep studies showed that Set significantly decreased the apnea index during sleep, in comparison with both pair-fed and Veh-treated animals (Figure 5, H–J). Thus, the stimulating effect of Set on hypercapnic sensitivity and the treatment effect on SDB persisted during chronic use.

### MC4R^+^ neurons in the parafacial region, but not in the NTS, regulated breathing and HCVR without affecting metabolism.

**Figure 6:**
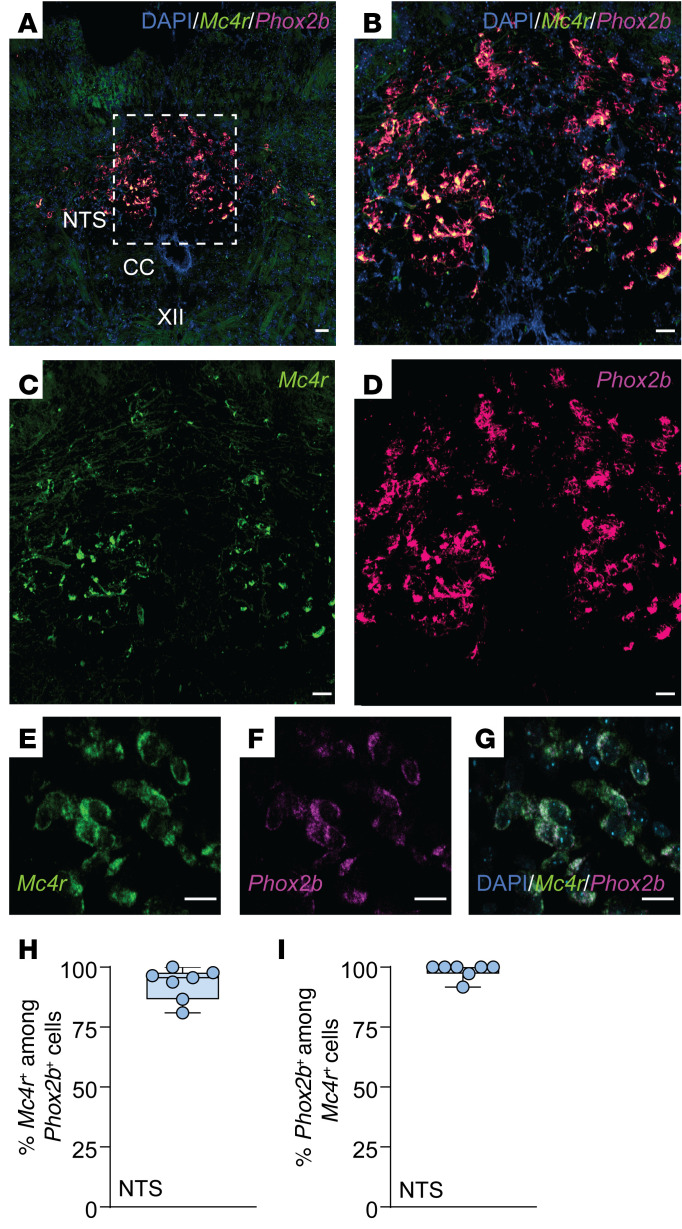
*Mc4r* is expressed in *Phox2b^+^* neurons of the NTS. (**A**–**G**) Lower-power (**A**) and higher-power (**B**–**G**) images of the NTS with DAPI, *Mc4r*, and *Phox2b* probes and merged images. (**H**) Percentage of *Phox2b^+^* cells expressing *Mc4r* in the NTS. (**I**) Percentage of *Mc4r^+^* cells expressing *Phox2b* in the NTS. CC, central canal; XII, hypoglossus nucleus. *n* = 7–8 mice. Scale bars: 50 μm (**A**); 30 μm (**B**–**D**); 20 μm (**E**–**G**).

**Figure 7:**
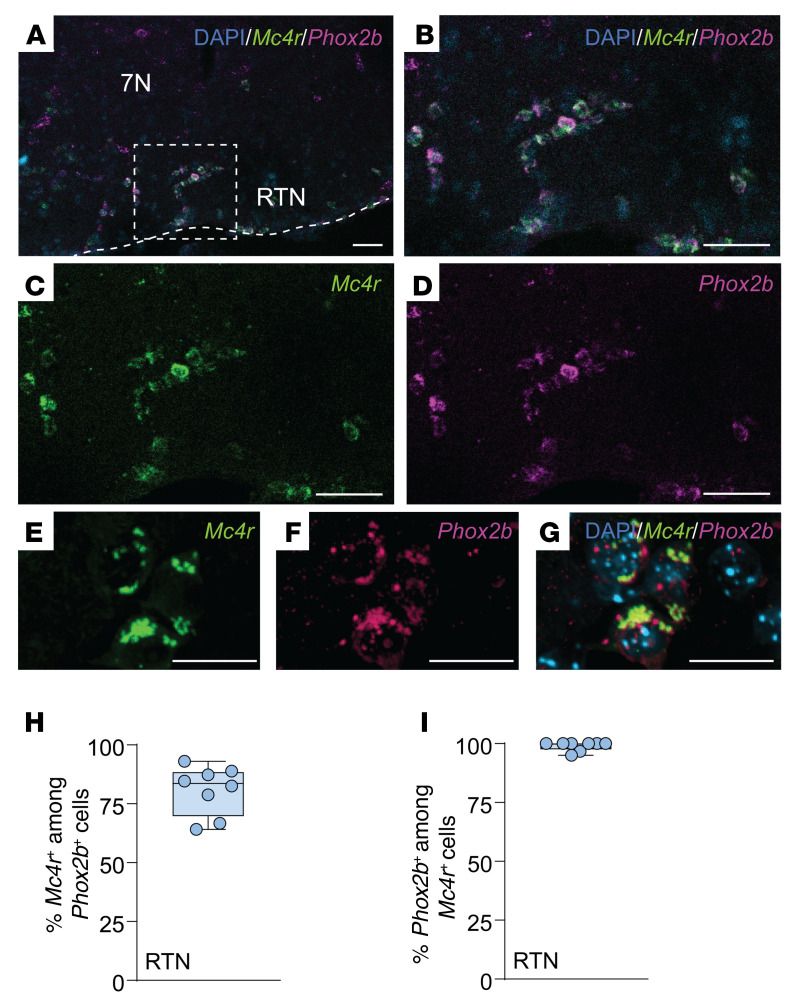
*Mc4r* is expressed in the *Phox2b^+^* neurons of the retrotrapezoid nucleus. (**A**–**G**) Lower-power (**A**) and higher-power (**B**–**G**) images of the retrotrapezoid nucleus (RTN) with DAPI, *Mc4r*, and *Phox2b* probes and merged images. (**H**) Percentage of *Phox2b^+^* cells expressing *Mc4r* in the RTN. (**I**) Percentage of *Mc4r^+^* cells expressing *Phox2b* in the RTN. 7N, facial nucleus. *n* = 7–8 mice. Scale bars: 50 μm (**A**–**D**); 15 μm (**E**–**G**).

**Figure 8:**
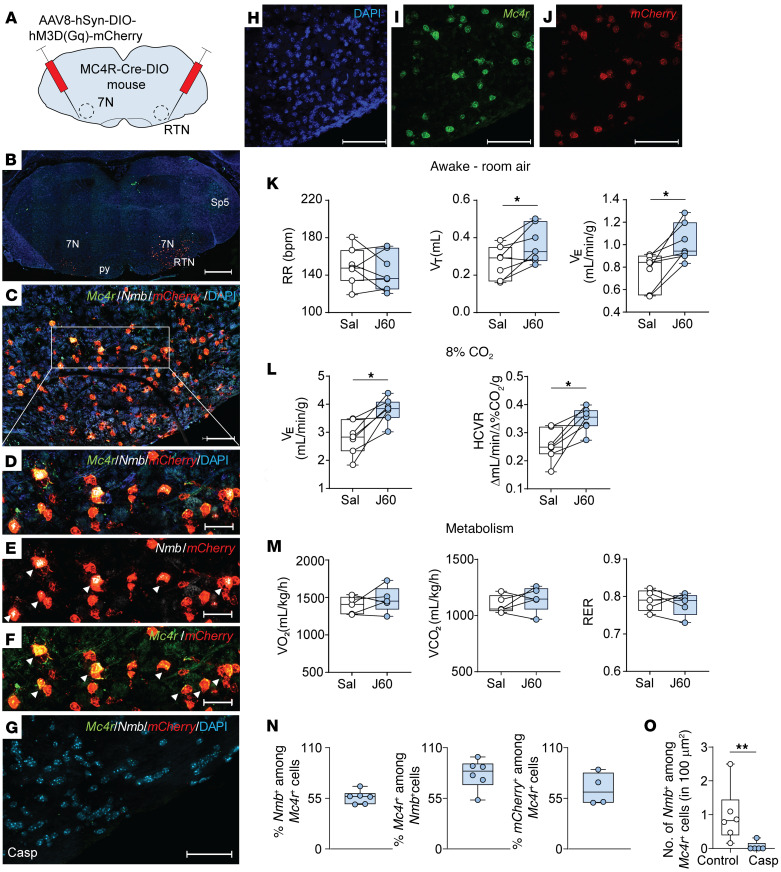
Chemogenetic stimulation of MC4R^+^ neurons in the RTN (ventral to the facial motor nucleus) increases *V*_E_ and the HCVR, but not metabolism. (**A**) *Cre*-dependent DREADDs AAV8-hSyn-DIO-hM3D(Gq)-mCherry was deployed in the RTN of *Mc4r-Cre* DIO mice. (**B**–**G**) In situ hybridization images. (**B**) Lower-power image of the brainstem containing RTN. (**C**) High-power image of the ventral surface of the brainstem containing *Mc4r*/*Nmb*/*mCherry*/DAPI. The outlined area is enlarged in **D**–**F**. The arrowheads point to *Nmb* and *mCherry* (**E**) and *Mc4r* and *mCherry* (**F**) colocalizations. (**G**) Histology in the presence of *Cre-*dependent caspase (AAV5-flex-taCasp3-TEVp) (Casp). Note a significant reduction in *Mc4r* and *Nmb*. (**H**–**J**) Individual in situ hybridization images showing DAPI in blue (**H**), *Mc4r* in green (**I**), and *mCherry* in red (**J**). (**K**–**M**) Upon stimulation with the DREADDs ligand J60, mice showed increases in *V*_T_ and *V*_E_ (**K**) and HCVR (**L**) without any effect on oxygen consumption (VO_2_), CO_2_ production (VCO_2_), or the respiratory exchange ratio (RER) (**M**) in comparison with saline (Sal). *n* = 5–7. *P ≤ 0.05, using the Wilcoxon’s matched-pairs, signed-rank test. (**N**) Percentage of colocalized cells. (**O**) Number of *Nmb^+^* among *Mc4r^+^* cells. **P < 0.01, using the Mann-Whitney *U* test (*n* = 5 – 6). 7N, facial motor nucleus. Sp5, spinal trigeminal nucleus. Scale bars: 500 μm (**B**); 100 μm (**C**); 50 μm (**D**–**F**); 30 μm (**G**); 100 μm (**H**–**J**).

*Phox2b* is one of the key markers of CO_2_-sensing neurons (65, 66). Therefore, we examined *Mc4r* and *Phox2b* mRNA coexpression in DIO male mice with OHS (Figures 6 and 7). We found that 93% and 81% of *Phox2b^+^* NTS and RTN neurons, respectively, expressed *Mc4r*, which may implicate a role of MC4Rs in respiratory chemosensitivity. Notably, nearly all *Mc4r^+^* cells in the NTS and RTN expressed *Phox2b*. Fifty-six percent of *Mc4r^+^* parafacial neurons also expressed *Nmb* (Figure 8, C–F and N), another marker of chemosensitivity, which confirms that a majority of these cells are CO_2_/H^+^-sensitive RTN neurons (68, 69). Eighty-one percent of *Nmb^+^* RTN neurons expressed *Mc4r* mRNA (Figure 8, C–F and N). Nevertheless, MC4R was not fully specific to the RTN, since 26% of *Mc4r^+^* neurons in the parafacial region expressed tyrosine hydroxylase and 40% expressed choline acetyltransferase, in catecholaminergic and cholinergic neurons, respectively, populations that are typically not involved in RTN chemosensitivity (65, 66, 68, 69) (Supplemental Figure 10).

Given the robust expression of *Mc4r* and *Phox2b* RNA in the parafacial region/RTN area and NTS, we examined whether MC4R^+^ neurons in these regions regulate CO_2_ sensitivity and metabolism. Four weeks after transfection of the parafacial region in *Mc4r-Cre* mice with AAV harboring *Cre*-dependent DREADDs, DREADDs (mCherry) or control (AAV expressing *Cre*-dependent mCherry without DREADDs) were deployed just below the facial motor nucleus, which is consistent with the RTN location (65) (Figure 8, A and B). DREADDs transfection in the parafacial region was effective, with 65% *Mc4r^+^* neurons expressing *mCherry*, and specific, since only 7.2% ± 3.0% of *mCherry^+^* neurons did not have detectable *Mc4r* mRNA by in situ hybridization (Figure 8, C–F, H–J, and N). In mice transfected with excitatory DREADDs, chemogenetic stimulation of MC4R^+^ neurons with the DREADDs ligand J60 increased baseline *V*_T_ and *V*_E_ (*P* < 0.05) (Figure 8K) and augmented the HCVR (*P* < 0.05) (Figure 8L) without any effect on metabolism (*P* > 0.05) (Figure 8M and Supplemental Figure 11). In contrast, J60 had no effect on breathing in mice transfected with control AAV to the RTN (Supplemental Figure 12). Thus, chemogenetic activation of MC4R^+^ RTN neurons is sufficient to stimulate baseline normocapnic breathing and HCVR.

**Figure 9:**
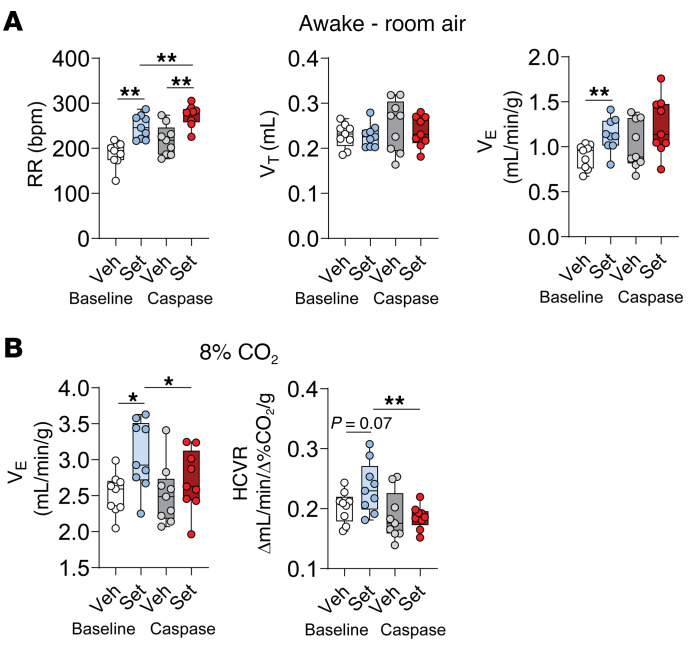
*Cre-*dependent caspase (AAV5-flex-taCasp3-TEVp) (Casp) administered to the RTN of *Mc4r-Cre* mice abolishes the effect of Set on the HCVR. Elimination of MC4R^+^ neurons in the RTN did not affect breathing at normocapnic conditions, and the stimulating effect of Set on RR was still present (**A**); but the effects of Set on *V*_E_ at 8% CO_2_ and HCVR were abolished (**B**). *n* = 9. *P ≤ 0.05, **P < 0.01, using the Wilcoxon’s matched-pairs, signed-rank test.

In order to examine whether respiratory effects of Set are mediated by the MC4R^+^ RTN neurons, we transfected *Mc4r-Cre* mice with AAV harboring *Cre*-dependent caspase to the parafacial region. The virus essentially eliminated CO_2_/H^+^-sensing *Mc4r^+^* *Nmb^+^* neurons in the parafacial region (Figure 8, G and O). Caspase did not affect breathing at normocapnic conditions, and the stimulating effect of Set on RR was still present after caspase transfection (Figure 9A). However, caspase treatment eliminated effects of Set on *V*_E_ at 8% CO_2_ and HCVR (*P* ≤ 0.05) (Figure 9B). Thus, the MC4R agonist Set stimulates HCVR by acting on MC4R^+^ neurons in the RTN.

**Figure 10:**
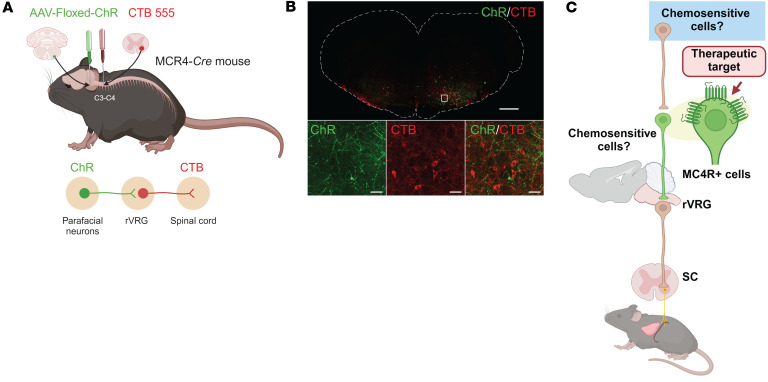
Parafacial MC4R^+^ neurons are synaptically connected to the rVRG neurons, which innervate phrenic motoneurons. (**A**) *Cre*-dependent ChR2-EYFP was deployed in the parafacial region of *Mc4r-Cre* mice, and the the C3–C4 segments of the spinal cord were injected with cholera toxin B (CTB) Alexa Fluor 555. (**B**) Lower-power image shows extensive density of MC4R^+^ green fibers surrounding CTB^+^ red cells (top panel). Higher-power images of the same region show (from left to right) MC4R^+^ fibers, CTB^+^ neurons, and a merged image showing that green MC4R^+^ fibers enmesh the red CTB^+^ neurons (bottom panels). Scale bars: 100 μm (top panel) and 50 μm (bottom panels). (**C**) Conceptual paradigm describing how MC4R^+^ neurons stimulate breathing. SC, spinal cord.

Next, we explored whether MC4R^+^ parafacial neurons innervate respiratory motoneurons. We expressed EYFP in the MC4R^+^ parafacial neurons and injected the retrograde tracer CTB into the phrenic motor nucleus in the C3–C4 segments of the spinal cord. We detected extensive projections of MC4R^+^ parafacial neurons to retrogradely labeled CTB^+^ rVRG neurons (Figure 10, A and B). These findings suggest that MC4R^+^ parafacial neurons likely monosynaptically project to the rVRG neurons (Figure 10C). Four weeks after transfection of the NTS in *Mc4r-Cre* mice with AAV containing *Cre*-dependent DREADDs, DREADDs (mCherry) was deployed bilaterally throughout the NTS (Supplemental Figure 13, A–F). Chemogenetic stimulation of MC4R^+^ NTS neurons did not affect *V*_E_ under room air conditions (*P* > 0.05) (Supplemental Figure 13G) and the HCVR (*P* > 0.05) (Supplemental Figure 13H). Surprisingly, chemogenetic activation of NTS MC4R^+^ neurons trended to reduce VO_2_ and VCO_2_ in the light phase (Supplemental Figure 14).

## Discussion

Currently, there is no effective pharmacotherapy for OHS. Our study shows that targeting MC4R receptors with a single dose of the MC4R agonist Set increased respiratory rate and enhanced minute ventilation in DIO male and female mice. However, respiratory effects were sex dependent. In males, Set upregulated VO_2_ and VCO_2_, but the robust increases in minute ventilation were out of proportion to modest increases in the metabolic rate. In male mice, Set augmented the hypercapnic ventilatory response and abolished SDB, eliminating apneas, attenuating oxyhemoglobin desaturations, and increasing minute ventilation during sleep and wakefulness. The beneficial effect of Set on the HCVR and apneas persisted throughout a 2-week course of treatment. In contrast, in DIO females, Set induced hyperventilation in proportion to increased metabolism. MC4R was abundantly expressed in the *Phox2b*^+^ neurons of the NTS and the parafacial region. Chemogenetic stimulation of the MC4R^+^ neurons in the NTS had neither respiratory nor metabolic effects. In contrast, chemogenetic stimulation of the parafacial MC4R^+^ neurons robustly increased the HCVR without any metabolic effect, suggesting that MC4R regulates respiratory control and metabolism via different neuronal populations. A majority of *Mc4r^+^* parafacial neurons expressed *Nmb*, suggesting that they were, in fact, CO_2_/H^+^-sensitive RTN neurons (68, 69). Finally, the parafacial MC4R^+^ neurons extended axonal projections to the rVRG neurons that receive projections from the cervical spine, suggesting that MC4R^+^ neurons innervate the phrenic nucleus in a disynaptic manner.

Set augmented the HCVR and treated sleep hypoventilation and SDB in DIO mice. Suppressed hypercapnic sensitivity is a key feature of the pathogenesis of OHS (1, 30, 78). Therefore, augmentation of the HCVR may significantly improve OHS morbidity and mortality. We have previously demonstrated that the DIO mouse (29) reveals a typical phenotype of OHS with daytime hypercapnia in the arterial blood gas and hypoventilation with recurrent hypopneas and apneas during sleep (28, 29, 31, 79). Our findings suggest that MC4R agonists could be effective in human OHS. One concern is that Set is used for weight loss only in patients with mutations in the POMC/leptin/MC4R pathways (50, 52–54), but not in the most common polygenic form of obesity. However, Set increases energy expenditure in polygenic obesity (80). Most importantly, our DREADDs data showed that activation of the MC4R receptor in the parafacial region augmented the HCVR in DIO mice without any effect on metabolism, which suggests that respiratory and metabolic effects of MC4R agonists are distinct and independent. Therefore, it is conceivable that Set will be effective in patients with the most common polygenic/environmental OHS. Moreover, we have shown that Set is a potent respiratory stimulant not only in obese but also in lean male mice. These data imply that Set can be considered for treatment of SDB and hypoventilation syndromes that are not related to obesity, such as drug-induced respiratory depression or genetic hypoventilation syndromes.

Set increased respiratory rate and minute ventilation in DIO females. However, in contrast to male data, these increases appear to be proportional to increases in VCO_2_ and were not accompanied by enhanced hypercapnic sensitivity. We have previously shown that, unlike males, DIO female mice do not develop hypoventilation (81) and demonstrate a much more robust HCVR compared with DIO males (82). We hypothesize that sex difference in respiratory responses to Set may be related to the ceiling effect in DIO female mice, which do not develop OHS.

We have shown that *Mc4r* mRNA is colocalized with *Phox2b* mRNA in the areas of the medulla involved in the control of breathing, the NTS and the parafacial region of the rostral ventrolateral medulla, which includes the RTN. We have not seen colocalization of *Mc4r* with *Phox2b* abundantly present in other brain regions. Polyalanine expansion mutations in *Phox2b* lead to impaired hypercapnic sensitivity and congenital central hypoventilation syndrome (67). The pH-sensitive *Phox2b*^+^ NTS neurons contribute to the hypercapnic sensitivity (83). *Phox2b* has been traditionally recognized as a marker of CO_2_-sensing RTN neurons (65, 66). MC4R expression in the parafacial region was not specific to the RTN, since fractions of MC4R^+^ neurons expressed tyrosine hydroxylase, a marker of catecholaminergic neurons, and choline acetyltransferase, a marker of cholinergic neurons, which are absent in the RTN (65). However, our in situ hybridization data showed that 56% of parafacial MC4R^+^ neurons expressed a highly specific marker of RTN CO_2_/H^+^-sensing neurons, *Nmb*, whereas 81% of *Nmb^+^* RTN neurons were also *Mc4r^+^* (68, 69). Therefore, mechanistic physiological evidence was needed to show that NTS and parafacial MC4R^+^ neurons indeed modulate HCVR.

Chemogenetic activation of the MC4R^+^ neurons in the NTS had no effect on the CO_2_ response in vivo, despite abundant levels of DREADDs expression. Our findings do not completely exclude the role of NTS MC4R^+^ neurons in control of breathing, which may require more robust levels of DREADDs expression or differential activation of subpopulations of NTS neurons. Nevertheless, our data provide no evidence that the site of respiratory effects of MC4R agonists is in the NTS. In contrast, chemogenetic activation of MC4R^+^ neurons in the parafacial region robustly enhanced the HCVR, suggesting their role in CO_2_ sensing. Moreover, caspase-driven elimination of parafacial MC4R^+^ neurons abolished effects of Set on HCVR. Taken together, our data implicate the parafacial MC4R^+^ neurons as a site of respiratory effects of Set and other MC4R agonists. Furthermore, parafacial MC4R^+^ neurons projected to the rVRG neurons, which were retrogradely labeled by CTB injections to the C3–C4 segments of the spinal cord containing phrenic motoneurons and, therefore, could be respiratory premotor neurons. Given the anatomic location of parafacial MC4R^+^ neurons and their role in the HCVR and colocalization with *Phox2b* and *Nmb* (Figures 7 and 8), our data suggest that parafacial MC4R^+^ neurons are chemosensitive RTN neurons.

Our data showed that Set treated hypoventilation both in the absence and in the presence of upper airway obstruction. Ninety percent of OHS patients have OSA (15), characterized by loss of upper airway muscle tone during sleep; therefore the effect of Set on IFL breathing indicates that Set relieved upper airway obstruction during sleep. The increase in hypercapnic sensitivity may increase genioglossus activity and dilate the upper airway obstruction, treating OSA (84, 85). It has yet to be tested whether parafacial MC4R^+^ neurons project to rVRG neurons that innervate not only phrenic but also hypoglossal motoneurons.

Our study had several limitations. First, breathing during sleep was fully evaluated only in an acute experiment after a single dose of Set. Nevertheless, 2-week treatment enhanced the HCVR as much as a single-dose regimen (Figures 1 and 5) and decreased the apnea rate, independent of weight loss, which indicates that chronic treatment of SDB is likely to be effective. Second, although differences in respiratory effects of Set between male and female mice are likely related to the ceiling effect in DIO females with robust hypercapnic chemosensitivity at baseline, more detailed exploration of sex differences in MC4R signaling and respiratory effects of MC4R agonists is needed. Third, elimination of MC4R^+^ RTN neurons using *Cre*-dependent caspase expression in the parafacial region suppressed the HCVR, but did not abolish effects of Set on normocapnic breathing, which suggests that Set has other sites of action in addition to the RTN. Fourth, Set decreased sleep time and impaired sleep architecture. The relationships between respiratory and sleep effects of MC4R agonists should be assessed during chronic treatment trials. Finally, our data suggest that MC4R agonists target neurons given that DREADDs expression is driven by the neuron-specific synapsin promoter. However, astrocytes also play a role in chemosensitivity (86, 87), which was not examined in our study.

In conclusion, we have shown that MC4R agonists increase the HCVR, increase minute ventilation during sleep and wakefulness, and abolish apneas and oxyhemoglobin desaturations during sleep, treating sleep-disordered breathing in obesity, and that the respiratory effects of MC4R agonists occur in the parafacial region, possibly in the RTN. Our study identifies parafacial MC4R^+^ neurons as a potential drug target in OHS and possibly in other central hypoventilation syndromes.

## Methods

### Sex as a biological variable.

Our study included both male and female mice and analyzed sex-based differences between them.

In total, 51 male DIO (#380050) and 13 male lean (#000664) mice on the C57BL/6J background were purchased from The Jackson Laboratory. Thirteen female C57BL/6J mice were purchased at 8–10 weeks of age from The Jackson Laboratory and fed a high-fat diet (5.4 kcal/g, 58.4% of kcal from fat; TD 03584, Teklad). DIO male mice were purchased at 18 weeks of age and fed with a high-fat diet. All the animals were used when they reached 20–24 weeks of age. Twenty-five *Mc4r-Cre* mice were purchased from The Jackson Laboratory (#008330) and fed with a high-fat diet. Water and food were available ad libitum. Mice were housed under standard environmental conditions (24°C–26ºC in a 12-hour light/12-hour dark cycle, 9 am–9 pm lights on).

### Experimental design.

Protocol 1 studied the effects of a single dose of setmelanotide (Set; 1 mg/kg intraperitoneally [i.p.] vs. vehicle [Veh]) on the hypercapnic ventilatory response (HCVR) and metabolism (oxygen consumption [VO_2_] and carbon dioxide production [VCO_2_]) in a randomized crossover study. Mice were treated either with Veh or with Set, followed by HCVR and metabolic measurements. A week later, mice that received Veh were treated with Set and vice versa, and studies were repeated.

Protocol 2 studied the effects of a single dose of Set (1 mg/kg i.p.) on breathing during sleep. Mice were randomized for the successive treatments with Set or Veh in a crossover trial, 1 week apart.

Protocol 3 studied the effect of 2-week daily Set (1 mg/kg i.p.), Veh, or pair feeding on ventilation awake and on the HCVR and apnea during sleep in a randomized 2-arm study.

Protocol 4 studied whether *Mc4r* and *Phox2b* are colocalized in the NTS and RTN using RNA in situ hybridization protocol in frozen sections of DIO C567BL/6J mice.

Protocol 5 studied the effects of chemogenetic stimulation of MC4R^+^ neurons in the location consistent with the RTN or the NTS on minute ventilation (*V*_E_), the HCVR, and metabolism in *Mc4r-Cre* DIO mice. *Mc4r-Cre* mice were transfected with *Cre*-dependent DREADDs to the RTN or NTS areas according to the stereotactic coordinates. Four weeks later, mice were randomized for sequential treatment with the DREADDs ligand J60 (0.1 mg/kg i.p. vs. saline, 1 week apart, cross-over design).

Protocol 6 studied the effects of loss of function of MC4R^+^ neurons in the RTN. We transfected DIO *Mc4r-Cre* mice in the RTN with AAV carrying *Cre-*dependent caspase and measured the effect of Set on room air ventilation and HCVR before and after caspase treatment.

### Hypercapnic ventilatory response (HCVR).

Mice were acclimated to the barometric plethysmography before the experiments, in accordance with our former studies (88, 89). HCVR measurements were performed at 30°C during the light phase. Mice were acclimated with a continuous-bias flow controlled with mass flow controllers in room air for 30 minutes. On the day of the experiment, C57BL/6J DIO and lean mice were randomized to Set (RM-493, Selleck Chemicals; 1 mg/kg i.p. in 2% heat-inactivated mouse serum plus 0.5% DMSO) versus Veh (mouse serum plus 0.5% DMSO). *Mc4r-Cre* DIO mice transfected with DREADDs viruses delivered to the RTN or NTS underwent a randomized crossover study of the DREADDs ligand J60 (0.1 mg/kg) (79) versus saline 1 week apart. They were challenged with a gas mixture of 8% CO_2_, 21% O_2_, and balanced in nitrogen. *Mc4r-Cre* DIO mice transfected with AAV carrying *Cre-*dependent caspase delivered to the RTN underwent a randomized crossover study of Set versus Veh 1 week apart before and after caspase treatment. For exposure, room air was switched to the hypercapnic mixture. The analyses were done after 1 minute of exposure when the ventilation reached a plateau. Tidal volume (*V*_T_), respiratory rate (RR), and minute ventilation (*V*_E_) were measured in mice at baseline (room air), and HCVR was determined in each animal by the slope of the relationship between *V*_E_ and inspired CO_2_ (0%–8%) during wakefulness via linear least-squares regression analysis.

### Metabolic measurements.

Metabolic studies were done in a randomized crossover design. Mice were housed in individual Comprehensive Laboratory Animal Monitoring System (CLAMS) units (Oxymax series, Columbus Instruments) for a 24-hour acclimation period followed by 24 hours of continuous recordings starting at 10:00 am (89, 90). The CLAMS units were sealed and equipped with O_2_ electrochemical sensors, CO_2_ infrared sensors, and infrared beam movement sensors. Every 11 minutes, consumed O_2_ (VO_2_) and produced CO_2_ (VCO_2_) were collected, and measurements were used to calculate the respiratory exchange ratio. An array of infrared photo beams that surrounded the metabolic cage was used to record the mouse activity as the mice moved. The locomotor activity was quantified by the counts of infrared beam interruptions. Total horizontal and vertical beam breaks were summed and presented as motor activity. Mice had free access to food and water during the measurements. Metabolic cages were kept on a 12-hour light/12-hour dark cycle (7 am–7 pm lights on) and at a consistent environmental temperature of 29°C–30°C.

### Sleep studies.

For polysomnography, mice were head-mounted with electroencephalogram (EEG) and nuchal electromyogram (EMG) electrodes as we have previously described (28, 29, 31, 79, 89, 91). All surgeries were done aseptically. For head-mount implantation, mice were deeply anesthetized with isoflurane 1%–2% and placed in the stereotaxic system (model 963 with 923-B Head Holder, David Kopf Instruments). Body temperature was kept at about 37°C using a heating pad. After the absence of withdrawal reflex to a firm pinch of paw and tail, a longitudinal midline incision was performed on their skull, and a head-mount (8201, Pinnacle Technology) was implanted for EEG and EMG recordings. In brief, 4 EEG electrode screws were placed bilaterally in the frontal and parietal bones, and 2 insulated EMG leads were tunneled subcutaneously and placed over the nuchal muscle posterior to the skull. Dental acrylic (Lang Dental) was used to secure the head-mount in place. Wounds were sutured, and all mice received 0.03 mg/kg of buprenorphine i.p. and were housed in a recovery chamber under heat. Mice were monitored and received additional buprenorphine if signs of distress or pain were observed.

After 1-week recovery and acclimation to the whole-body barometric plethysmography chamber (WBP, Buxco) and the pulse oximetry neck collar, the mice were randomized to Set versus Veh treatment, which was administered at 9:30 am followed by sleep studies from 10 am to 4 pm. For polysomnography, we used our modified WBP chamber system, which allowed us the measurement of tidal airflow and sleep/wake state continuously (29, 71, 90). The chamber was calibrated to allow high-fidelity *V*_T_ and airflow signals. Body weight and rectal temperature were measured at the beginning and end of the sleep study. During the recordings, the chamber was humidified at 90% and kept at about 29°C, while a slow leak allowed atmospheric pressure equilibrium. The WBP’s reference chamber filtered out ambient noise from the pressure signal acquired by a transducer. Positive and negative pressure sources were used in series with mass flow controllers (Alicat Scientific) and high-resistance elements to generate a continuous-bias airflow through the animal chamber while maintaining a sufficiently high time constant. To calculate the tidal airflow from the plethysmography chamber pressure signal, the Drorbaugh and Fenn equation was used (92). This formula requires the measurement of mouse rectal temperature, chamber temperature, room temperature, relative humidity, and chamber gas constant, calculated by use of the chamber pressure deflection of a known volume injection.

All signals were digitized at 1,000 Hz (sampling frequency per channel) and recorded in LabChart 7 Pro (version 7.2, ADInstruments). The sleep/wake state was scored visually in 5-second epochs based on standard criteria of EEG and EMG frequency content and amplitude, as previously described (29, 89–91, 93, 94). Wakefulness, NREM sleep, and REM sleep were observed consistently throughout the experiments. Wakefulness was identified by a trained observer as low-amplitude, high-frequency (~10 to 20 Hz) EEG waves, and high levels of EMG activity in comparison with the sleep states. NREM sleep was scored as epochs with high-amplitude, low-frequency (~2 to 5 Hz) EEG waves with EMG activity considerably less than during wakefulness. REM sleep was characterized by low-amplitude, mixed-frequency (~5 to 10 Hz) EEG waves, and muscle atony. Breathing parameters were analyzed from periods of NREM sleep sampled periodically at 20-second stretches every half hour throughout the total recording time and from all REM sleep periods. The demarcation of the beginning and end of inspiration and expiration was done using custom software for subsequent calculations of timing and amplitude parameters for each respiratory cycle. Sleep efficiency was calculated as total sleep time divided by recording time after sleep onset.

Scoring investigators were blinded to experimental conditions. We used each breath’s respiratory characteristic to describe maximal inspiratory airflow (*V*_I_max) and components of minute ventilation (*V*_E_). We developed an algorithm using the airflow and respiratory effort signals to determine whether a breath was classified as inspiratory airflow limited, defined by an early inspiratory plateau in airflow while effort continued to increase (29, 93, 94). *V*_I_max, RR, *V*_T_, and *V*_E_ were reported separately for wakefulness, and for unobstructed and IFL breathing during NREM and REM sleep.

Apneas were scored manually as ≥90% reduction in airflow for 2 or more breath cycles or ≥0.7 seconds based on average RR at baseline (89). The apnea index was calculated by division of the number of apneas by the total sleep time (in hours). The oxyhemoglobin desaturation index (ODI) was quantified as ≥4% oxyhemoglobin desaturations from the baseline for at least 2 breaths (28, 29).

### Viral vector and retrograde tracer administration.

Male obese *Mc4r-Cre* mice were transfected with the excitatory *Cre*-dependent DREADDs viruses [AAV8-hSyn-DIO-hM3D(Gq)-mCherry, Addgene, 44361-AAV8; 4 × 10^12^ vg/mL] delivered bilaterally to the RTN or NTS; or AAV carrying *Cre-*dependent caspase (AAV5-flex-taCasp3-TEVp, Addgene, 45580; ≥7 × 10^12^ vg/mL) bilaterally to the RTN. Viral vector administration was done as previously described (90, 91). Briefly, mice were anesthetized with isoflurane for induction (2%–3% in the closed chamber), and after the absence of withdrawal reflex to a firm pinch of the tail they were set within the Kopf stereotaxic apparatus with mouse adapter and isoflurane vaporizer. Afterward, anesthesia was continued with 1%–2% isoflurane. A longitudinal midline incision was performed, and the underlying fascia was gently removed. One hundred twenty-five nanoliters of DREADDs and caspase vectors were delivered bilaterally using pre-pulled glass micropipettes (Sutter) to the RTN area using the following stereotactic coordinates: –1.4 mm caudal and ±1.4 mm lateral from the animal’s lambda and 4.6 mm ventral to cerebellum surface at 3 sites spanning the rostro-caudal length of the facial nucleus. For NTS injection we used the following coordinates: 7.60 to 7.80 mm anterior-posterior, 0.40 mm medial-lateral bilateral, and 4.80 to 5.00 mm dorso-ventral from the animal’s bregma in a separate group of mice (91). Buprenorphine (0.03 mg/kg) was administered at the end of surgery to minimize distress. After a 4-week recovery period, mice underwent a randomized crossover study of the DREADDs ligand J60 versus saline 1 week apart.

*Mc4r-Cre* mice (*n* = 4), 5 days of age, were transfected with *Cre*-dependent channelrhodopsin (ChR2)-EYFP [AAV1.EF1a.DIO.hChR2(H134R)-EYFP.WPRE.hGH, Penn Vector Core] to the parafacial region. Stereotaxic surgeries in neonatal pups were performed as described previously (95, 96). Briefly, animals, anesthetized with hypothermia, were mounted in a stereotactic apparatus with a neonatal adapter (Stoelting). The skull was exposed, and a small burr hole was made to position a pipette (VWR International) containing viral vector. Viral vector (30 nL) was injected unilaterally at the following coordinates: 3.1–3.2 mm posterior, 0.4 mm lateral, and 3.3–3.6 mm ventral relative to lambda. Four weeks later, these animals were anesthetized with isoflurane, as described above, and mounted in a stereotaxic apparatus. A dorsal midline incision was made at the level of the cervical spinal cord, and the muscles were reflected laterally. A retrograde tracer, cholera toxin subunit B conjugated to Alexa Fluor 555 (CTB; 100 nL; Invitrogen), was injected unilaterally into the phrenic motor nucleus of the cervical spinal cord (C3–C4) at the following coordinates: 0.9 mm from the dorsal surface of the spinal cord, and 0.7 mm lateral to the spinal midline. After 72 hours, animals were deeply anesthetized and sacrificed. Coronal slices of the medulla were made and examined using a fluorescence confocal microscope.

### RNAscope.

DIO C57BL/6J mice were deeply anesthetized with 1%–2% isoflurane and promptly perfused transcardially using ice-cold phosphate-buffered saline (PBS; 0.01 M, pH 7.4) solution followed by 4% paraformaldehyde in PBS. Brains were removed, postfixed in the same fixative for 4 hours, and cryoprotected in 30% sucrose overnight at 4°C. Brains were sectioned (14 μm) on a cryostat (HM 560, Thermo Fisher Scientific), and sections were stored at –80°C. RNA in situ hybridization protocol was performed on frozen sections using the standard RNAscope Assay (ACDBio, catalog 323100). The RNA probes of *Phox2b* (catalog 407861), *Mc4r* (catalog 319181), *ChAT* (catalog 408731), *Nmb* (catalog 459931), *Th* (catalog 317621), and *mCherry* (catalog 431201) were designed and synthesized by ACDBio. Images were taken by a Zeiss LSM 700 microscope (Johns Hopkins Multiphoton Imaging Core) and analyzed using ImageJ/Fiji software.

### Confocal imaging and image analysis.

Expression of the AAV8-hSyn-DIO-hM3D(Gq)-mCherry virus was confirmed by positive expression of mCherry in the NTS and RTN. For the immunofluorescent analysis of the brain, mice were anesthetized with 1%–2% isoflurane and promptly perfused transcardially using ice-cold PBS (0.01 M, pH 7.4) solution followed by 4% paraformaldehyde in PBS. Brains were removed, kept for 4 hours in 4% paraformaldehyde, and cryoprotected in 30% sucrose overnight at 4°C. Afterward, tissues were embedded in Tissue-Tek O.C.T. Compound (catalog 4583, Tissue-Tek) and frozen on dry ice. Coronal sections of the frozen brains were cut into 30 μm slices using a cryostat, and tissue was stored at –20°C before further processing. Slides were kept at room temperature for 5 minutes and then rehydrated with PBS. Sections were covered with mounting medium with DAPI (4,6-diamidino-2-phenylindole; Vectashield, Vector Laboratories). Images from the different experimental groups were captured and then examined under a Zeiss Cell Observer Spinning Disk Confocal microscope. Localization of DREADDs in the brainstem was confirmed by visualization of mCherry expression. MC4R^+^ parafacial neurons projecting to CTB^+^ rostral ventral respiratory group (rVRG) were detected using a Zeiss 980 confocal system.

### Statistics.

Data were analyzed using Prism version 10.4.1 (GraphPad Software Inc.). D’Agostino & Pearson and Shapiro-Wilk tests were used to test normality. We used a mixed-effects model to examine whether the factors affected the results and whether there was interaction overall. One-way ANOVA, paired 2-tailed *t* test, or Wilcoxon’s matched-pairs, signed-rank test was used to compare differences within groups. Mann-Whitney *U* test was used to compare differences between groups. *P* levels less than or equal to 0.05 were considered statistically significant. Descriptive statistics were obtained from the summary tables of each statistical analysis and are mentioned in the text as the mean ± SEM. Graphically, data were plotted using box plots showing individual mice.

In order to analyze a complex longitudinal data set in the chronic Set treatment experiment, we fitted robust linear models (RLMs) to outcome data representing REM, NREM, and total sleep. Because error distribution for each outcome variable was right-skewed and comprised zeros, we added a negligible positive constant to all values before log-transforming in order to satisfy assumptions of homoscedastic variance. We fitted RLMs as functions of group, categorized as Set, Veh, and pair feeding. We performed 2-tailed hypothesis tests for the group coefficient with 5% alpha. We reported model parameter estimates and visualized results on the original, back-transformed scale (i.e., not on the log scale). We fitted RLMs and visualized RLM predictions using the R statistical computing environment (R Core Team, 2021).

### Study approval.

We conducted all animal experiments in accordance with protocols approved by the Johns Hopkins University Animal Care and Use Committee (protocol MO19M191) and by the George Washington University Animal Care and Use Committee (protocol A2023-014).

### Data availability.

All data reported in this paper will be shared upon reasonable request. Values for all data points in the graphs are provided in the Supporting Data Values file.

## Author contributions

MRA, NRW, OA, MAR, DM, and VYP designed research studies. MRA, NRW, OA, MAR, FAD, JLDD, JX, OD, and SBF conducted experiments. MRA, NRW, OA, MAR, FAD, JLDD, JX, OD, SBF, and SML analyzed data. MRA, MNW, RSA, DM, and VYP acquired funding. DM and VYP performed project administration. MRA, DM, and VYP wrote the original draft. MRA, NRW, OA, MAR, FAD, JLDD, JX, OD, SBF, SML, JSB, MNW, RSA, DM, and VYP reviewed and edited the manuscript.

## Supplementary Material

Supplemental data

Supporting data values

## Figures and Tables

**Table 1:**
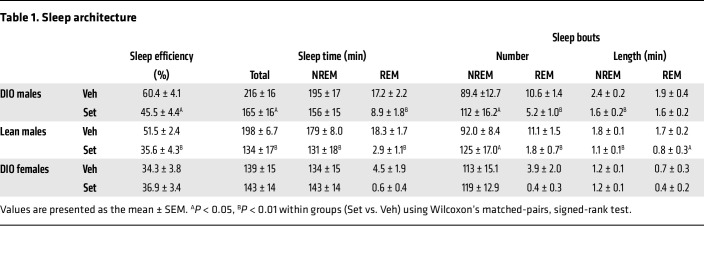
Sleep architecture

## Version 1 04/15/2025

In-Press Preview

## Version 2 06/16/2025

Electronic publication
