# Supplementary material for: Targeting melanocortin 4 receptor to treat sleep-disordered breathing in mice
Source: J Clin Invest. 2025 Apr 15;135(12):e177823. doi: 10.1172/JCI177823 (PMC12165796; doi:10.1172/JCI177823)
Supplement: Supplemental data [file jci-135-177823-s179.pdf]

Supplementary material

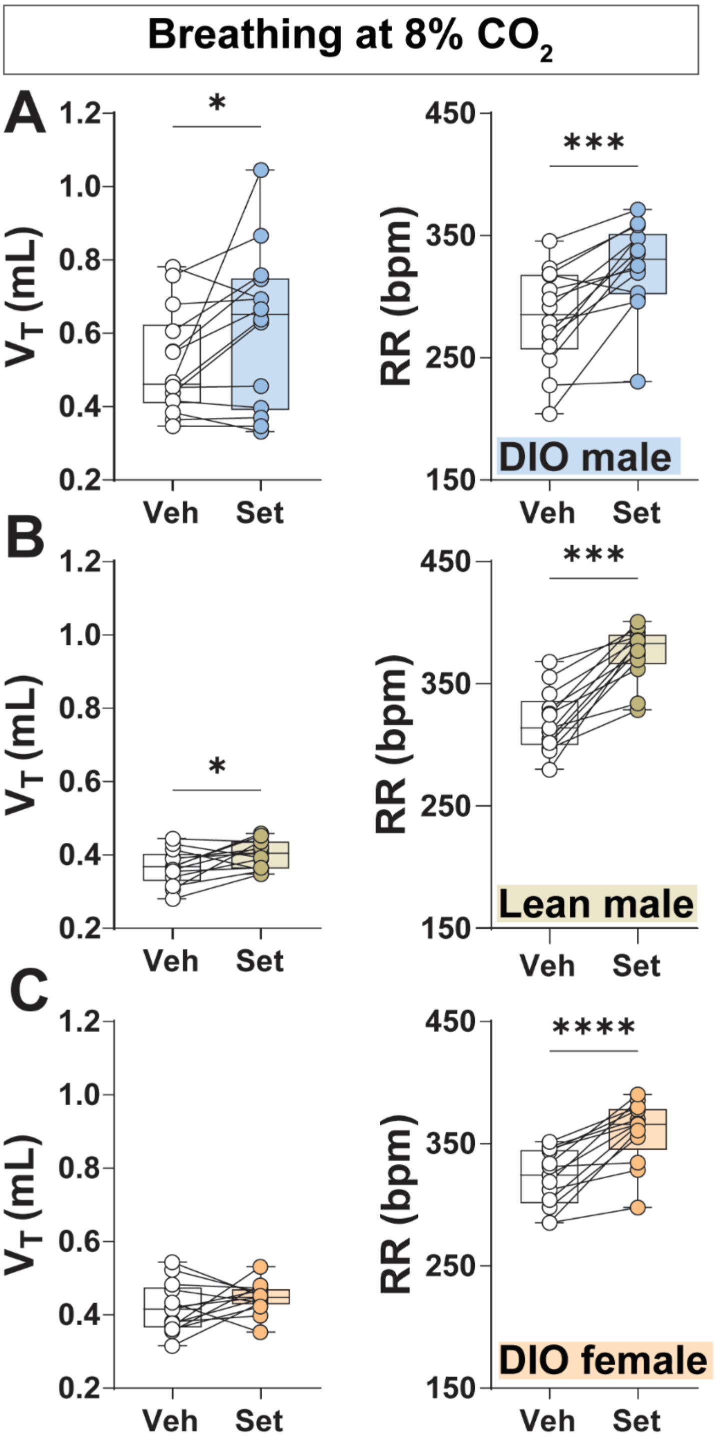

Figure S1: Individual and grouped data showing the effects of the vehicle (Veh) and setmelanotide (Set) on tidal volume (V_T) and respiratory rate (RR) in awake **(A)** diet-induced obese (DIO) male mice (N = 14), **(B)** lean male mice (N = 13) and, (**C**) DIO female mice (N = 13) at 8% of inspirited CO_2. * *P* ≤ 0.05, *** *P* < 0.001 and, **** *P* < 0.0001 using Wilcoxon matched-pairs signed rank test or paired t test.

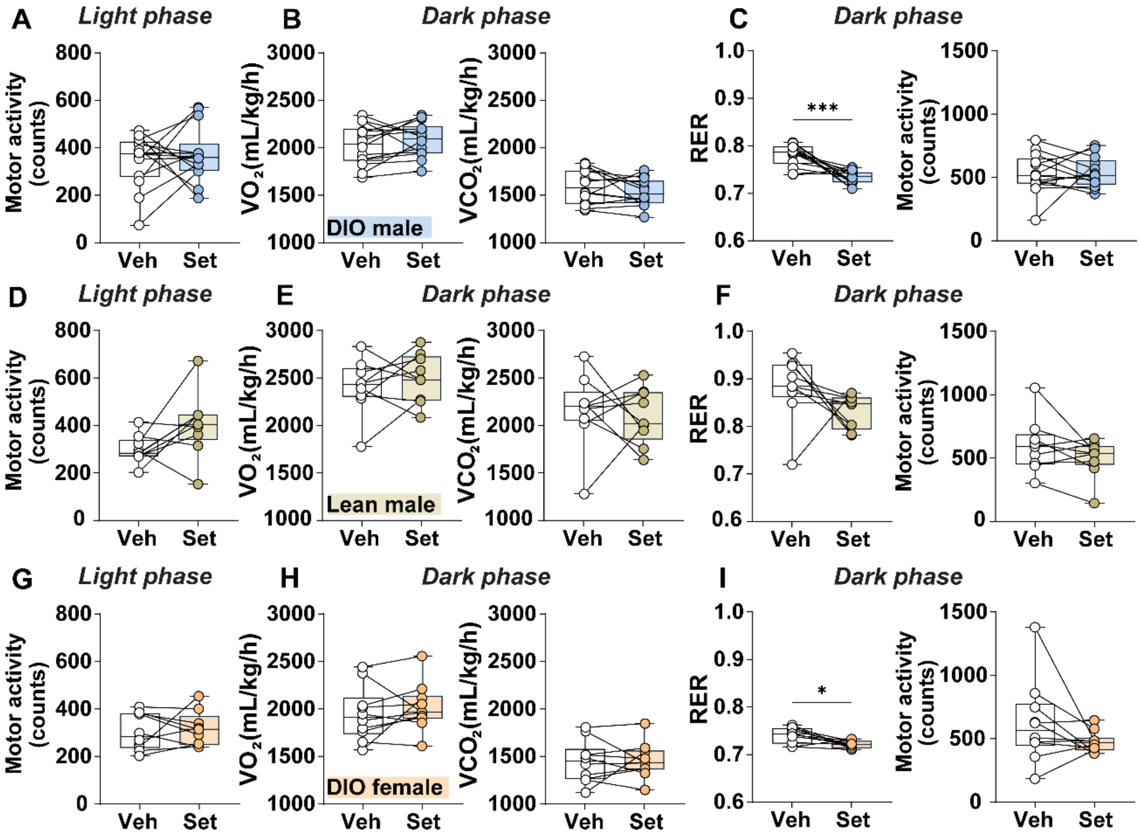

Figure S2: Individual and grouped data showing the effects of the vehicle (Veh) and setmelanotide (Set) on **(A)** total motor activity in the light phase, **(B)** total oxygen consumption (VO_2) and total carbon dioxide production (VCO_2), (**C**) respiratory exchange ratio (RER) and total motor activity in dark phase in diet-induced obese (DIO) male mice (N = 14). **(D)** total motor activity in the light phase, **(E)** VO_2 and VCO_2, (**F**) RER and total motor activity in dark phase in lean male mice (N = 9). **(G)** total motor activity in the light phase, **(H)** VO_2 and VCO_2, (**I**) RER and total motor activity in dark phase in DIO female mice (N = 10). * $P \leq 0.05$ and *** $P < 0.001$ using Wilcoxon matched-pairs signed rank test.

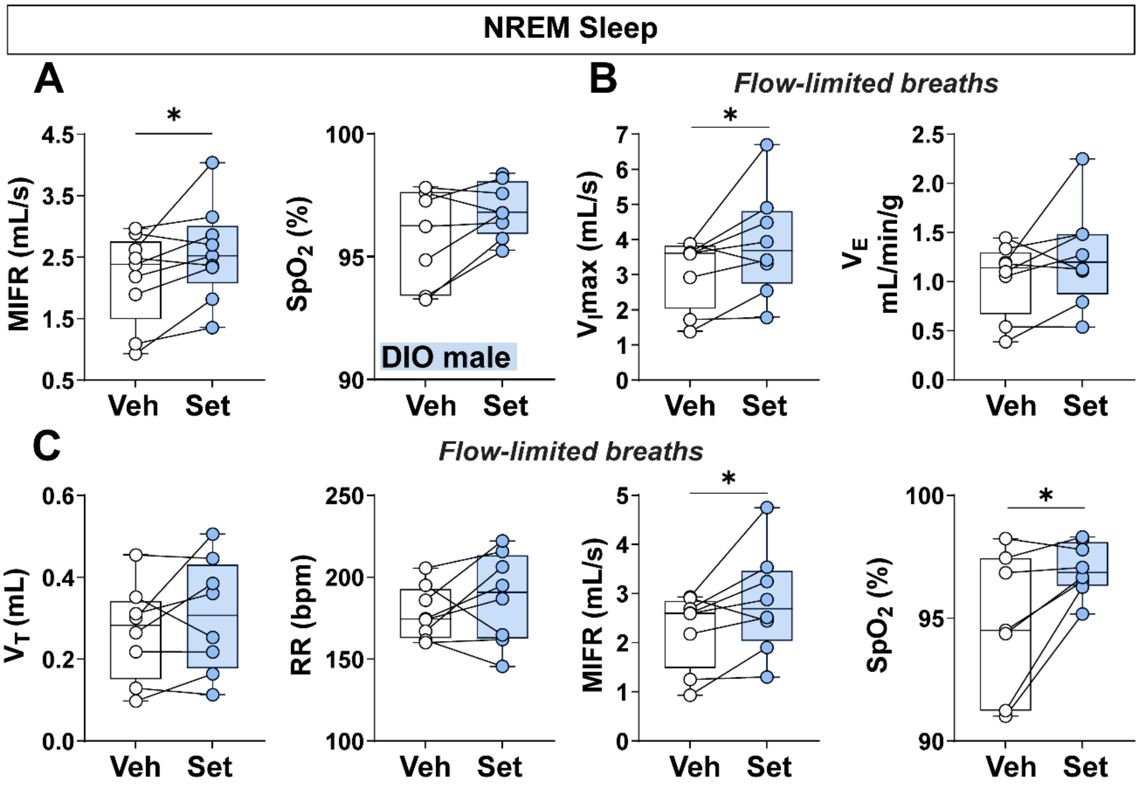

Figure S3: Individual and grouped data showing effects of the vehicle (Veh) and setmelanotide (Set) on **(A)** mean inspiratory flow rate (MIFR) and oxygen saturation (SpO_2), during non-flow limited breathing in non-rapid eye movement (NREM) sleep in diet-induced obese (DIO) male mice. **(B)** Maximal inspiratory flow ($V_I$max), minute ventilation (V_E), **(C)** tidal volume (V_T), respiratory rate (RR), MIFR, and SpO_2 in flow-limited breathing in NREM sleep in diet-induced obese (DIO) male mice. (N = 8-9). * $P \leq 0.05$ using Wilcoxon matched-pairs signed rank test.

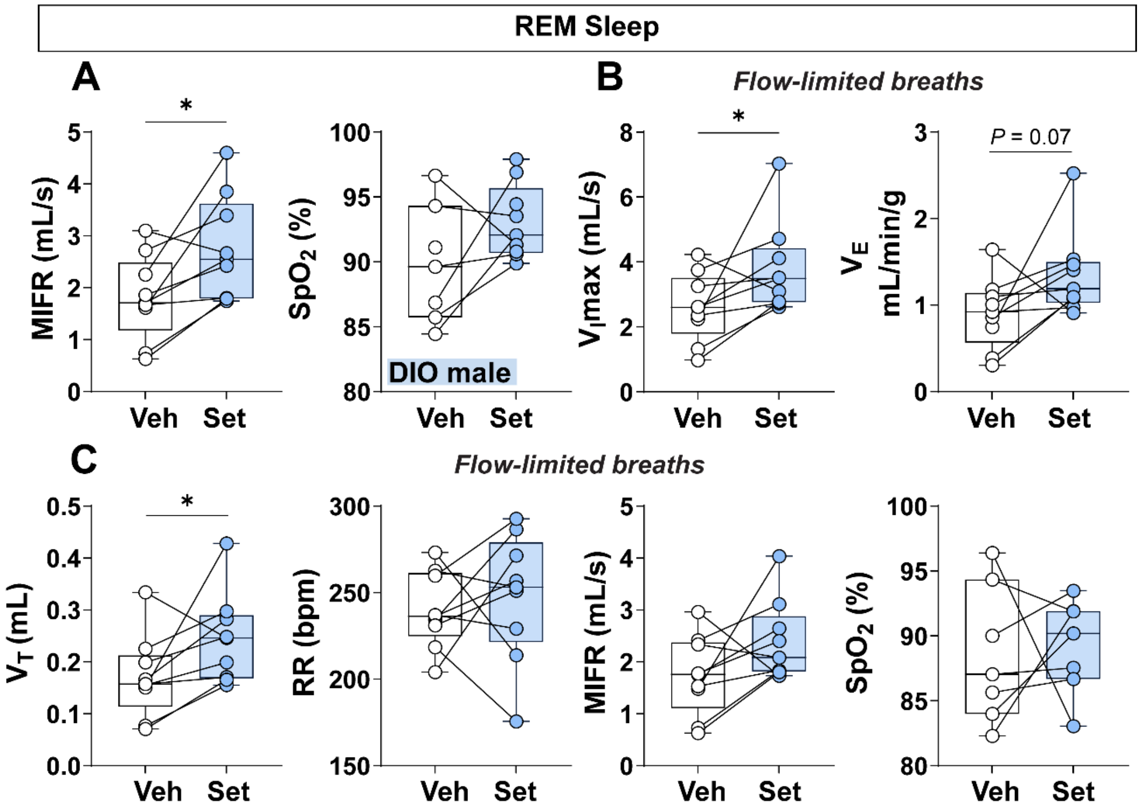

Figure S4: Individual and grouped data showing effects of the vehicle (Veh) and setmelanotide (Set) on **(A)** mean inspiratory flow rate (MIFR) and oxygen saturation (SpO_2), during non-flow limited breathing in rapid eye movement (REM) sleep in DIO male mice. **(B)** Maximal inspiratory flow (V_Imax), minute ventilation (V_E), **(C)** tidal volume (V_T), respiratory rate (RR), MIFR, and SpO_2 in flow-limited breathing in REM sleep in diet-induced obese (DIO) male mice. (N = 7-9). * $P \leq 0.05$ using Wilcoxon matched-pairs signed rank test.

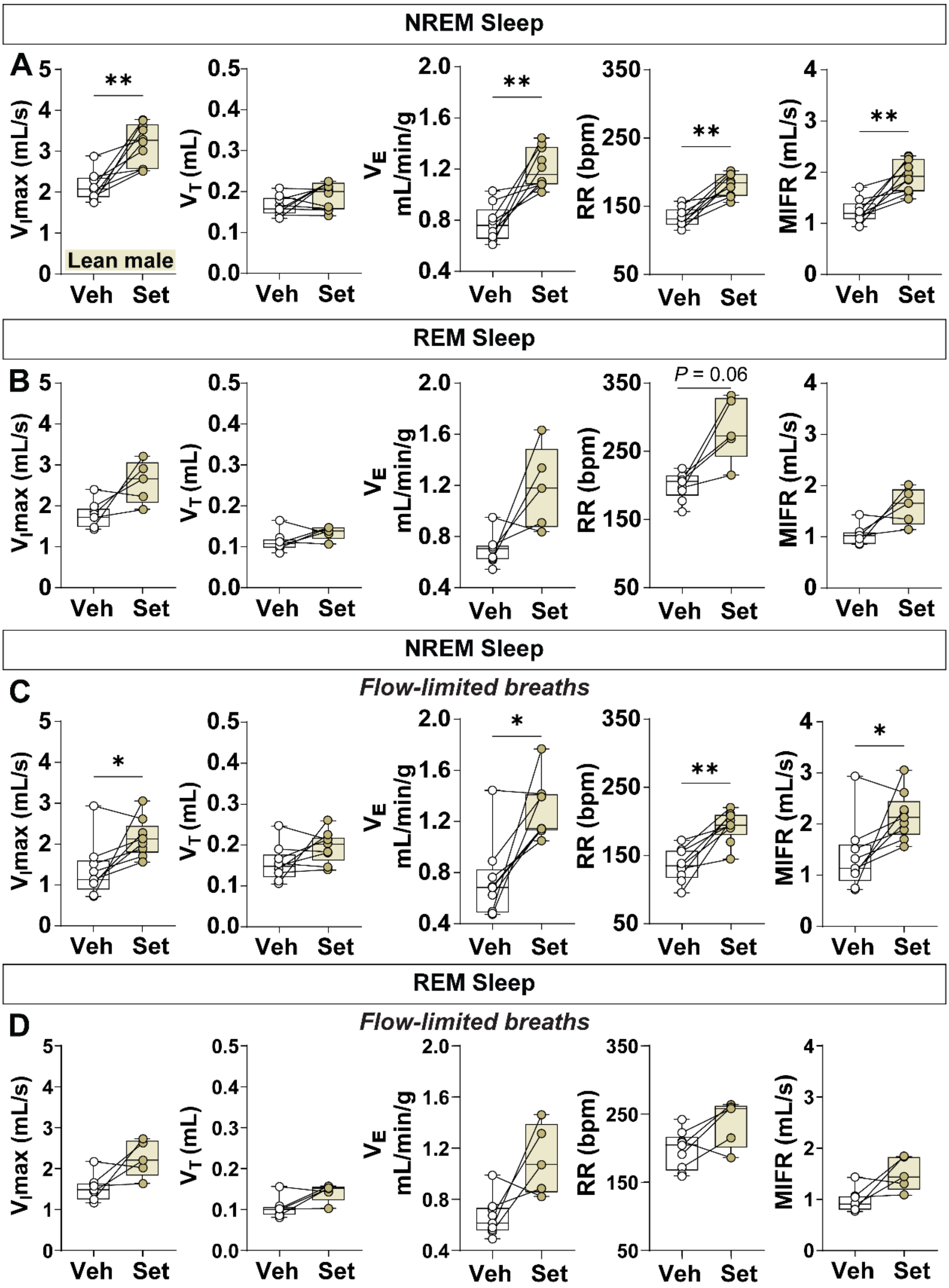

Figure S5: Individual and grouped data showing effects of the vehicle (Veh) and setmelanotide (Set) on maximal inspiratory flow ($V_I$max), tidal volume (V_T), minute ventilation (V_E), respiratory rate (RR) and, mean inspiratory flow rate (MIFR) during **(A)** non-flow limited breathing in non-rapid eye movement (NREM) sleep, **(B)** non-flow limited breathing in rapid eye movement (REM) sleep; **(C)** flow-limited breathing in NREM sleep and, **(D)** flow-limited breathing in REM sleep in lean male mice. (N = 5-9). * $P \le 0.05$ and ** $P < 0.01$ using Wilcoxon matched-pairs signed rank test.

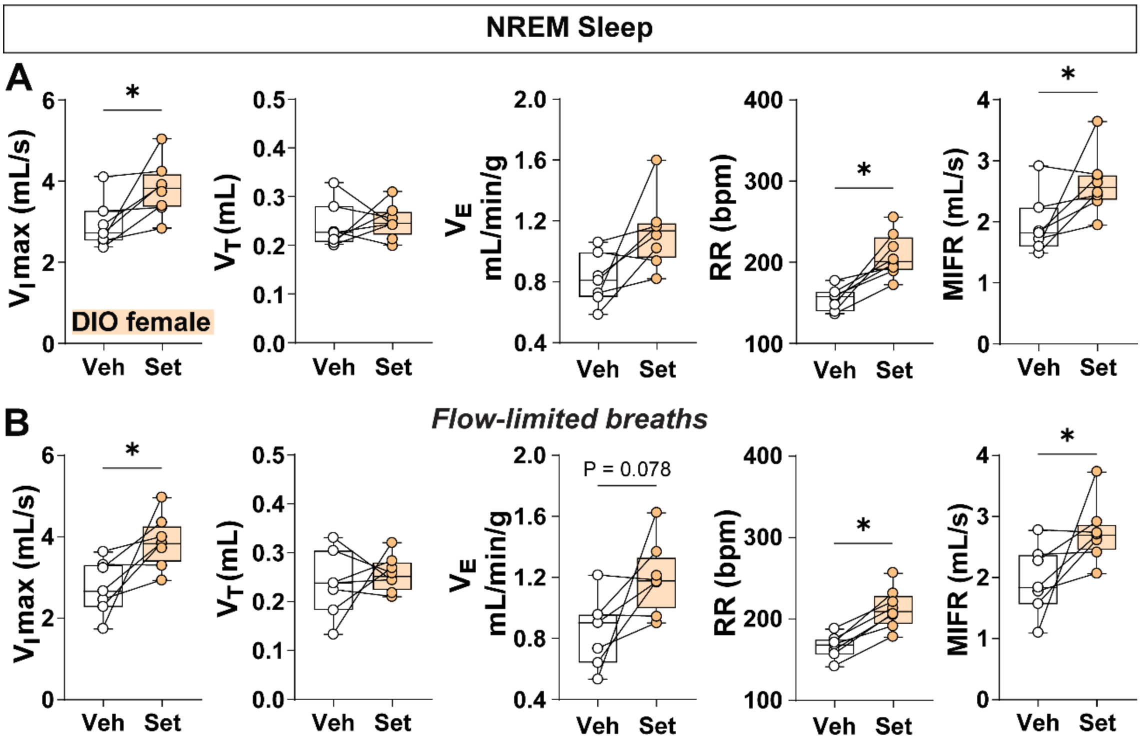

Figure S6: Individual and grouped data showing effects of the vehicle (Veh) and setmelanotide (Set) on maximal inspiratory flow ($V_I$max), tidal volume (V_T), minute ventilation (V_E), respiratory rate (RR) and, mean inspiratory flow rate (MIFR) during **(A)** non-flow limited breathing in non-rapid eye movement (NREM) sleep and, **(B)** flow limited breathing in NREM sleep in diet-induced obese (DIO) female mice. (N = 7-8). * $P \leq 0.05$ using Wilcoxon matched-pairs signed rank test.

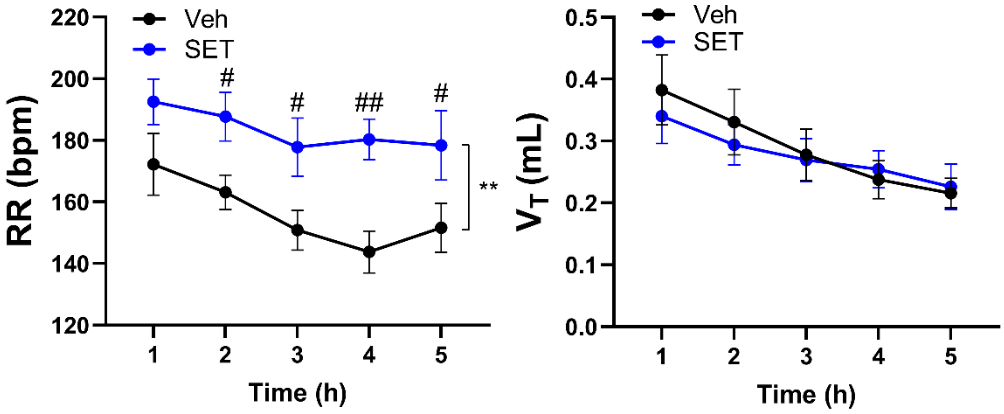

Figure S7: Time course of respiratory rate (RR) and tidal volume (V_T) during non–rapid eye movement sleep in diet-induced obese male mice. Means ± SEM. ** *P* ≤ 0.01 effect of tratment using mixed-effects model. # *P* ≤ 0.05 and ## *P* ≤ 0.01 using Wilcoxon matched-pairs signed rank test. (n = 11).

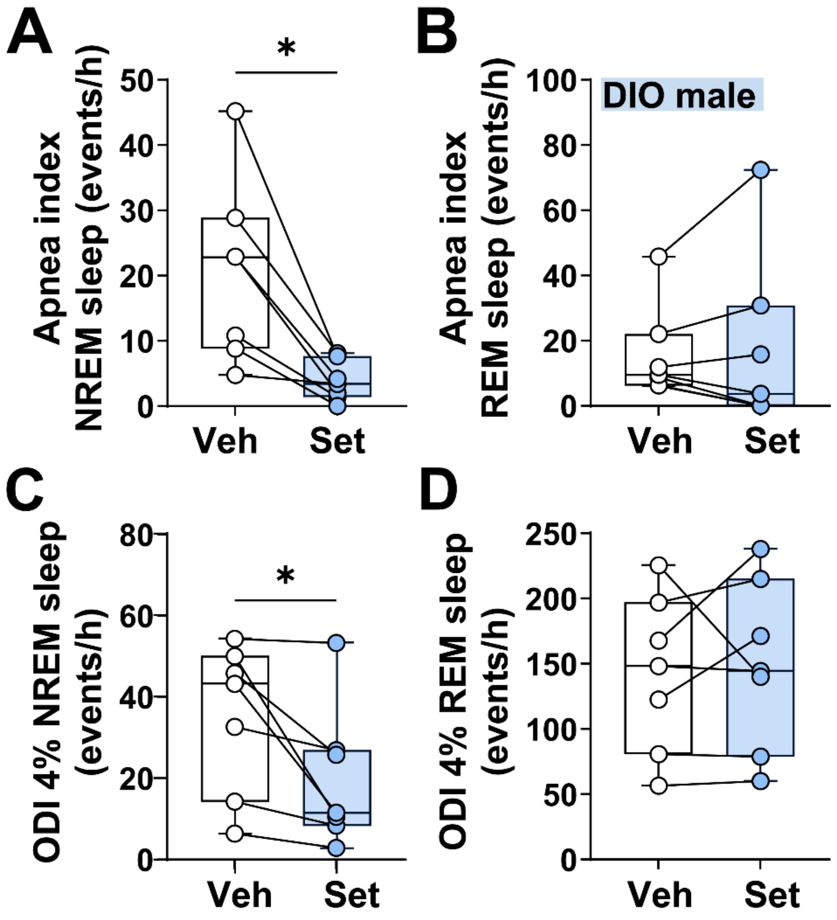

Figure S8: Individual and grouped data showing effects of the vehicle (Veh) and setmelanotide (Set) on apnea index during **(A)** NREM and **(B)** REM sleep in DIO male mice. ODI was defined as a number of oxyhemoglobin desaturations ≥4% from baseline per hour of **(C)** NREM sleep and **(D)** REM sleep in diet-induced obese (DIO) male mice. (n = 7). * *P* < 0.05 using Wilcoxon matched-pairs signed rank test.

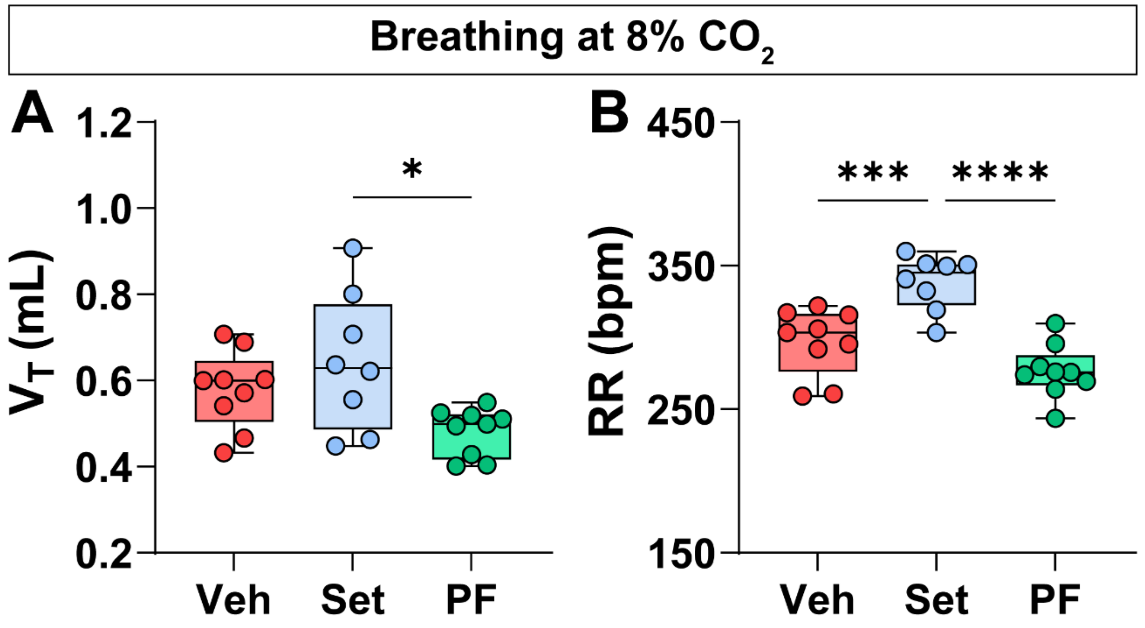

Figure S9: Individual and grouped data showing the effects of the vehicle (Veh, N = 9), setmelanotide (Set, N = 8) or pair-feeding (PF, N = 9) treatment for two weeks on **(A)** tidal volume (V_T) and **(B)** respiratory rate (RR) at 8% of inspirited CO_2 in awake diet-induced obese male mice. * $P \leq 0.05$, *** $P < 0.001$ and, **** $P < 0.0001$ using ona-way ANOVA with Tukey`s multiple comparisons test.

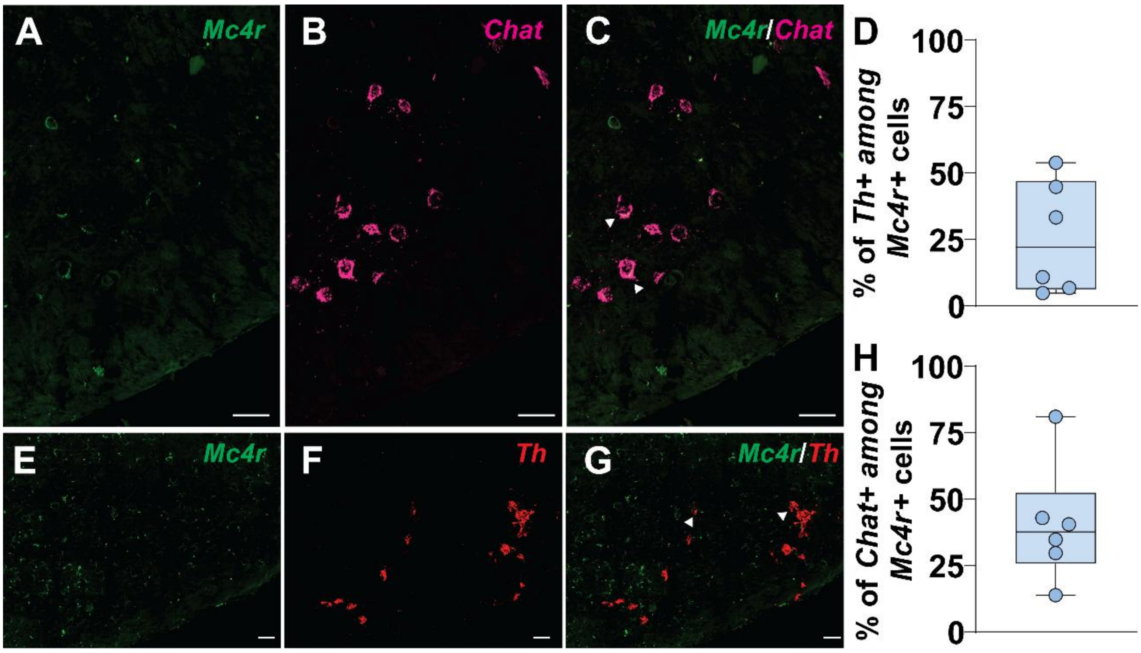

Figure S10: (A) *Mc4r* **(B)** *Chat* and **(C)** merged images of the retrotrapezoid nucleus (RTN). **(D)** % of *Th*+ among *Mc4r*+ cells (N = 6). **(E)** *Mc4r* **(F)** *Th* and **(G)** merged images of RTN. **(H)** % of *Chat*+ among *Mc4r*+ cells (N = 6). Arrows point at co-localization. Scale bar in A-C = 50 µm, in E-G = 50 µm

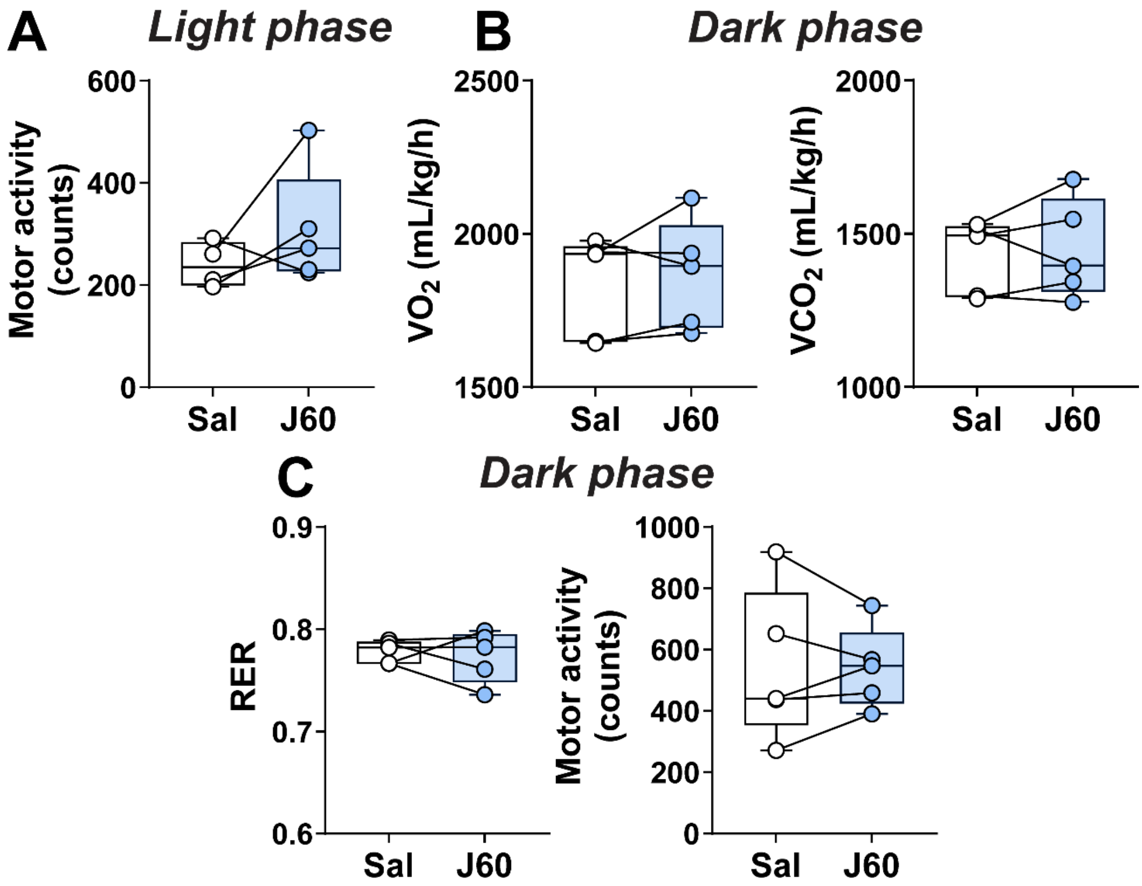

Figure S11: Individual and grouped data showing the effects of the saline (Sal) and the DREADD ligand, J60 on *Mc4r-Cre* diet-induced obese mice with *Cre*-dependent DREADD deployed in the RTN. **(A)** Total motor activity in the light phase. **(B)** Total oxygen consumption (VO_2) and total carbon dioxide production (VCO_2), (**C**) respiratory exchange ratio (RER) and total motor activity in dark phase (N = 4 - 5).

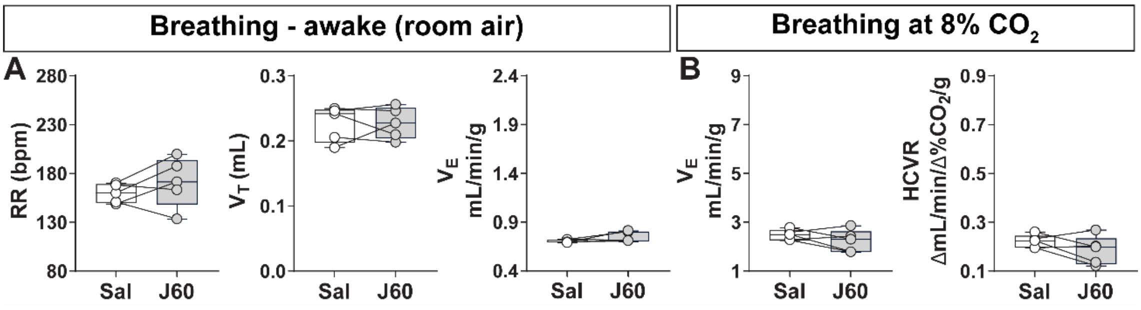

Figure S12: Individual and grouped data showing the effects of the saline (Sal) and the DREADD ligand, J60 on awake *Mc4r-Cr*e diet-induced obese male mice *Cre*-dependent Control virus deployed in the RTN. **(A)** respiratory rate (RR), tidal volume (V_T) and minute ventilation (V_E) under room air condition. **(B)** V_E at 8% of inspired CO_2 and hypercapnic ventilatory response (HCVR) (N = 5).

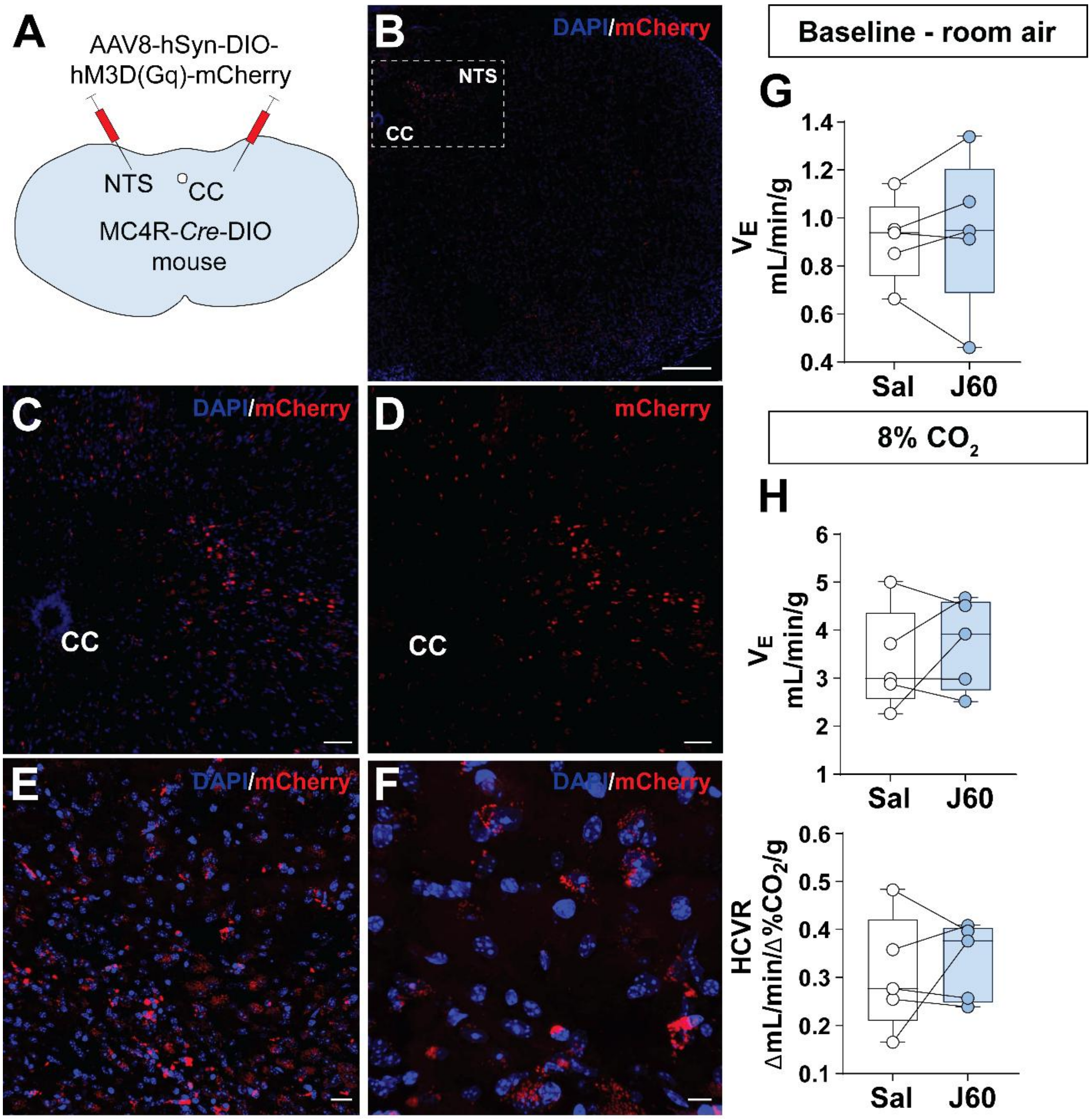

Figure S13: Chemogenetic stimulation of MC4R(+) neurons in the nucleus of the solitary tract (NTS) does not affect minute ventilation (V_E) or the hypercapnic ventilatory response (HCVR). **(A)** *Cre*-dependent DREADD AAV8-hSyn-DIO-hM3D(Gq)-mCherry was deployed in the NTS of *Mc4r-Cre* diet-induced obese male mice; **(B)** Lower power NTS images showing DAPI and mCherry; The outline are is enlanrged in **(C and D). (E and F)** Higher power imagens showing indivudual cells with DAPI-mCherry merge**;** Upon stimulation with the DREADD ligand J60, mice do not show increases in **(G)** minute ventilation (V_E) or **(H)** V_E at 8% of inspired CO_2 and hypercapnic ventilatory response (HCVR). (N = 5). Scale bar in B = 300 µm, C and D = 50 µm, E = 20 µm and F = 10 µm

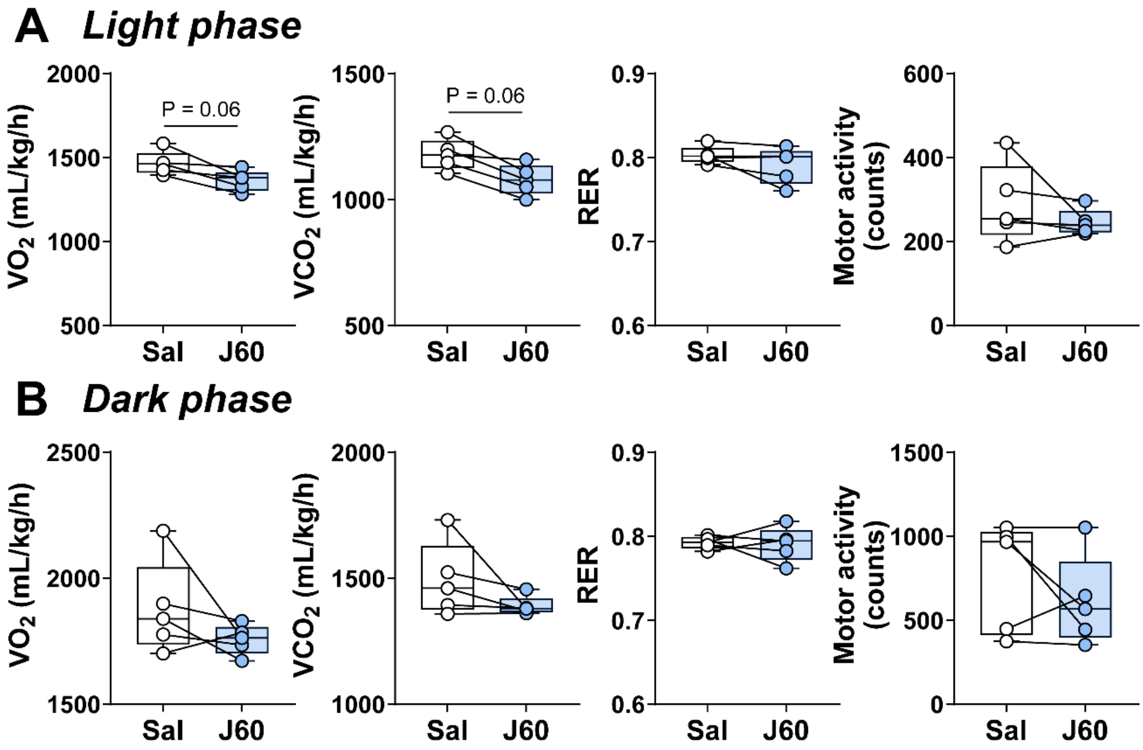

Figure S14: Individual and grouped data showing the effects of the saline (Sal) and DREADD ligand, J60 on *Mc4r-Cre* DIO male mice with *Cre*-dependent DREADD deployed in the NTS. **(A)** Total oxygen consumption (VO_2), total carbon dioxide production (VCO_2), respiratory exchange ratio (RER) and total motor activity in light phase. **(B)** VO_2, VCO_2, RER, and total motor activity in dark phase (N = 5).
